# WASH phosphorylation balances endosomal versus cortical actin network integrities during epithelial morphogenesis

**DOI:** 10.1038/s41467-019-10229-6

**Published:** 2019-05-16

**Authors:** Vasilios Tsarouhas, Dan Liu, Georgia Tsikala, Alina Fedoseienko, Kai Zinn, Ryo Matsuda, Daniel D. Billadeau, Christos Samakovlis

**Affiliations:** 1 Department of Molecular Biosciences, The Wenner-Gren Institute, Stockholm University, SE-106 91 Stockholm, Sweden; 2 SciLifeLab, SE-171 65 Stockholm, Sweden; 3 Department of Biochemistry and Division of Oncology Research, College of Medicine, Mayo Clinic, Rochester, MN 55905 USA; 4 Division of Biology and Biological Engineering, California Institute of Technology, Pasadena, CA 91125 USA; 5 Cardiopulmonary Institute, Justus Liebig University of Giessen, Aulweg 130, Giessen, 35392 Germany

**Keywords:** Actin, Endocytosis, Morphogenesis

## Abstract

Filamentous actin (F-actin) networks facilitate key processes like cell shape control, division, polarization and motility. The dynamic coordination of F-actin networks and its impact on cellular activities are poorly understood. We report an antagonistic relationship between endosomal F-actin assembly and cortical actin bundle integrity during *Drosophila* airway maturation. Double mutants lacking receptor tyrosine phosphatases (PTP) Ptp10D and Ptp4E, clear luminal proteins and disassemble apical actin bundles prematurely. These defects are counterbalanced by reduction of endosomal trafficking and by mutations affecting the tyrosine kinase Btk29A, and the actin nucleation factor WASH. Btk29A forms protein complexes with Ptp10D and WASH, and Btk29A phosphorylates WASH. This phosphorylation activates endosomal WASH function in flies and mice. In contrast, a phospho-mimetic WASH variant induces endosomal actin accumulation, premature luminal endocytosis and cortical F-actin disassembly. We conclude that PTPs and Btk29A regulate WASH activity to balance the endosomal and cortical F-actin networks during epithelial tube maturation.

## Abstract (web summary)

The regulation and coordination of F-actin networks are not well understood. Here, the authors show an antagonistic relationship between endosomal F-actin assembly and cortical bundle integrity during epithelial tube maturation that is regulated by kinase and phosphatase activity modifying WASH.

## Introduction

Endocytosis provides an interaction hub between epithelial tissues and their environment. It not only facilitates the uptake of essential nutrients but is also crucial for cell communication, as it determines the steady-state levels of cell surface receptors and their rapid clearance upon activation. Mechanisms of endocytosis have been traditionally elucidated by elegant studies in yeast and in cultured mammalian cells utilizing specific model cargoes^1^. More recently, systematic screens have identified distinct molecular networks required for different endocytic routes in cultured cells^2^, but we still lack a view on how endocytosis is regulated in vivo and on how it is integrated with other cellular activities and tissue physiology.

**Fig. 1:**
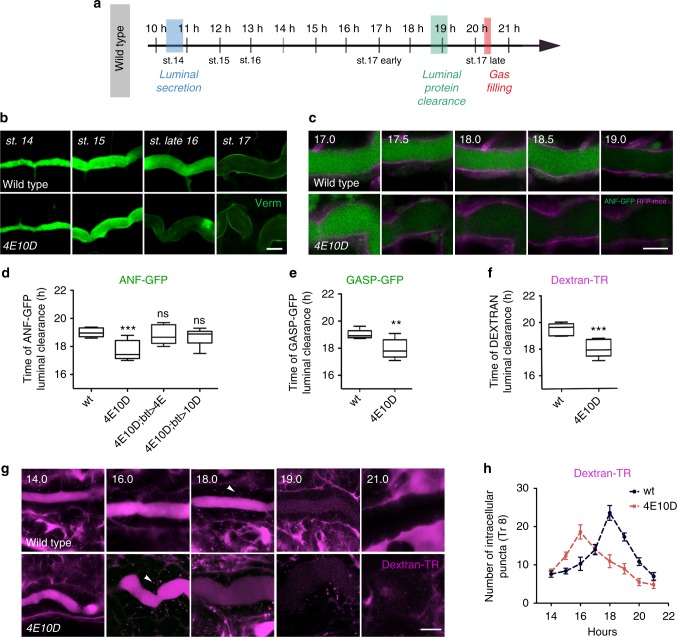
Ptp10D and Ptp4E control the precise timing of luminal protein clearance in a cell autonomous manner. **a** Schematic representation of the *Drosophila* airway maturation. The *x* axis depicts the time of embryo development in hours after egg laying (AEL) and the corresponding embryonic stages. The airway maturation steps luminal secretion, luminal protein clearance and gas filling are indicated. **b** Confocal images showing the tracheal dorsal trunk (DT) of stage 14–17 wild-type and *Ptp4EPtp10D* (*4E10D*) mutant embryos stained for the luminal protein Verm (green). **c** Confocal sections showing the tracheal DT of living wild-type and *Ptp4EPtp10D* mutant embryos expressing *btl>ANF-GFP* (green) and *RFP-moesin* (magenta). **d** Plots showing the average time (hours) of luminal ANF-GFP clearance in wild-type (*n* = 36), *Ptp4EPtp10D* (*n* = 43), *Ptp4EPtp10D*;*btl>ptp4E-GFP* (*n* = 28) and *Ptp4EPtp10D*;*btl>ptp10D* (*n* = 33) embryos, expressing *btl>ANF-GFP*. **e**, **f** Plots showing the average time (hours) of luminal *btl>GASP-GFP* (**e**) or Dextran-Texas Red (Dextran-TR, 10 kDa) clearance (**f**) in wild-type (GASP-GFP, *n* = 32; Dextran-TR, *n* = 18) and *Ptp4EPtp10D* mutants (GASP-GFP *n* = 36; Dextran-TR, *n* = 30). The boxplots (**d**–**f**) show the median (horizontal line) and the data range from 25th to 75th percentile. Bars present maxima and minima values. **P < 0.01. ***P < 0.0045. ns not significant (*P* > 0.05). Data collected from five independent experiments. Unpaired two tailed *t* test (**d**–**f**). **g** Confocal frames from live imaging showing the Dextran-TR luminal clearance in wild-type and *Ptp4EPtp10D* mutant embryos. Note that the premature luminal Dextran-TR clearance in *Ptp4EPtp10D* mutants is associated with earlier increase of intracellular puncta (16 h AEL) compared to wild type (18 h AEL) (arrowheads). **h** Graph representing the average number of intracellular Dextran-TR puncta in wild-type (*n* = 19) and *Ptp4EPtp10D* mutants (*n* = 30) during tracheal maturation. Data in each time point represent means from at least three independent experiments. Error bars represent the standard error of the means (s.e.m.). The time points **c**–**h** represent hours AEL. Scale bars, 10 μm

We use the *Drosophila* airways as an in vivo model to uncover regulatory mechanisms of apical endocytosis. Like mammalian lungs, the *Drosophila* respiratory system undergoes a precisely timed series of maturation events to convert the nascent branches into functional airways^3^. A massive wave of apical endocytosis is transiently activated in the airway epithelium at the end of embryogenesis to internalize luminal material and prepare the embryo for breathing (Fig. 1a). Mutations in several genes encoding endocytic components lead to clogged airways and larval lethality^3,4^.

Here, we define a regulatory circuit that controls the initiation of massive endocytosis and airway clearance by modulating the concurrent endosomal F-actin assembly and cortical actin bundle disassembly. It involves two type III receptor protein tyrosine phosphatases (PTPs), Ptp10D and Ptp4E, the non-receptor tyrosine kinase (non-RTK) Btk29A, and the actin nucleation-promoting factor WASH (Wiskott–Aldrich syndrome protein and SCAR homologue). Type III PTPs contain a single catalytic phosphatase domain in the cytoplasmic region and fibronectin type III-like domains in their extracellular region^5,6^. In *Drosophila*, Ptp10D and Ptp4E^7^ control airway tube shapes through negative regulation of epidermal growth factor receptor (EGFR) signalling^8^. In addition, Ptp10D antagonizes EGFR to promote tumour-suppressive epithelial cell competition^9^. *Drosophila* Btk29A is a Tec family non-receptor tyrosine kinase, regulating cytoskeletal rearrangements during epithelial development^10–12^ and wound healing downstream of RTK signalling^10,11,13–15^. The third component of the circuit, WASH, is critical for Arp2/3-induced F-actin polymerization and is required for endosomal membrane scission and cargo sorting^16–18^. Mammalian and *Drosophila* WASH proteins associate with members of the SHRC regulatory complex containing FAM21, Strumpellin, SWIP and CCDC53^17,19,20^. Contrary to WASP/N-WASP and WAVE, WASH activation is not fully understood. The Rho GTPase has been linked to WASH activation in *Drosophila*^21^ but is not sufficient to directly activate human WASH in vitro^18,22^.

We show that Btk29A and WASH are required for luminal clearance and that Btk29A induces WASH phosphorylation. Conversely, *Ptp4EPtp10D* mutants or Latrunculin B (LAT-B) treatment or overexpression of a dominant-negative form of the DAAM/Formin in the airways not only disassemble cortical actin bundles but also induce premature luminal clearance. A phosphomimetic WASH version is sufficient to induce endosomal actin accumulation and premature luminal endocytosis while it interferes with apical actin bundle integrity. We propose that the WASH phosphorylation status balances F-actin assembly between the endosomal and cortical F-actin networks to regulate the timing of luminal clearance and airway shape.

## Results

### Premature airway clearance in *Ptp4EPtp10D* mutants

During mid-embryogenesis, apical actin is organized in thick parallel bundles, running perpendicular to the tube axis of the *Drosophila* airways^23,24^. Concurrently to the initiation of luminal protein clearance at 18 h after egg laying (AEL), these structures are progressively lost (Supplementary Fig. 1a) suggesting a link between cytoskeletal remodelling and the initiation of apical endocytosis.

Genetic screens have identified hundreds of mutations blocking endocytosis and luminal clearance in *Drosophila* airways^3,4,25^. In sharp contrast to these, *Ptp4EPtp10D* double mutant embryos clear the luminal protein Verm earlier than wild type (WT; Fig. 1a, b). Live imaging of WT and mutant embryos expressing the luminal markers ANF-GFP and Gasp-GFP^3^ or carrying the fluid-phase endocytosis marker Dextran-Texas Red (Dextran-TR) in the airways showed that *Ptp4EPtp10D* mutants initiate and complete dorsal trunk (DT) clearance about 2 h earlier than WT (Fig. 1c–f). Precocious tube clearance in the mutants was accompanied by severe tube shape defects and an expansion of the apical cell surface visualized by α-catenin-GFP (Fig. 1b, and Supplementary Fig. 1b). At hatching, mutant airways failed to fill with gas and collapsed (Supplementary Fig. 1c). The premature clearance of ANF-GFP could be rescued by transgenic expression of either *Ptp4E* or *Ptp10D* in the tracheal tubes of the mutants indicating that the two PTPs act redundantly and cell autonomously (Fig. 1d).

We tested whether potential leakage through the epithelial diffusion barrier may underlie the precocious luminal protein clearance. However, injection of 10 kDa Dextran-TR into the haemocoel of *Ptp4EPtp10D* embryos showed that their airways were impermeable to the dye in contrast to *Mtf* mutants, which lack septate junctions (SJs)^26^ (Supplementary Fig. 1d). In addition, the localization of the paracellular SJ proteins Coracle (Cor) and Fasciclin III (FasIII), the adherens junction component DE-Cadherin (DEcad), the apical proteins (atypical protein kinase C (aPKC) and Uninflatable (Uif)) and the basal matrix component Viking remained unaffected in *Ptp4EPtp10D* embryos in comparison to WT (Supplementary Fig. 1e–j). Since luminal clearance depends on a wave of increased apical endocytosis^3^, these results argue that the premature luminal clearance phenotype in *Ptp4EPtp10D* embryos involves a precocious activation of massive endocytosis. This was further supported by the early, transient increase in internalized luminal 10 kDa Dextran-TR in the *Ptp4EPtp10D* airway cells (Fig. 1g, h). Concurrently with the precocious dextran endocytosis, the *Ptp4EPtp10D* DT tubes became tortuous and cystic (Fig. 1g).

**Fig. 2:**
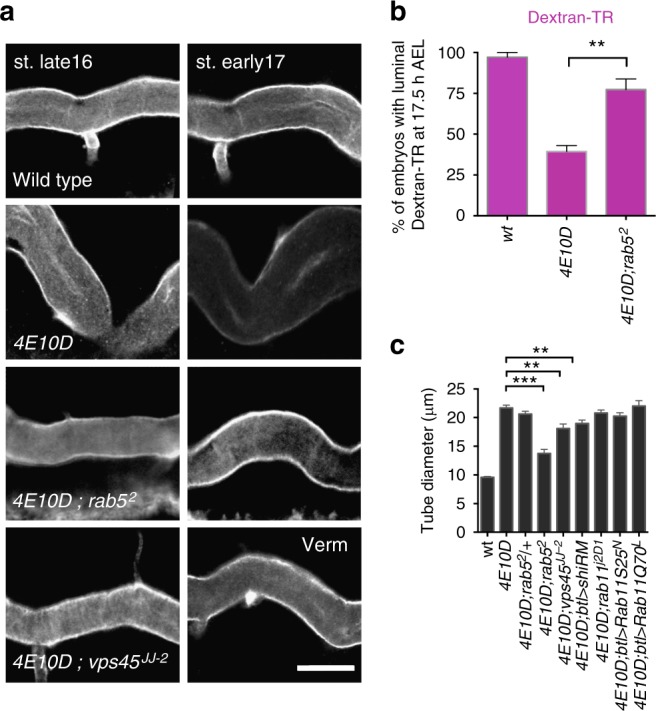
Reduction of endocytosis restores the *Ptp4EPtp10D* phenotypes. **a** Confocal images displaying the dorsal trunk (DT) of late-stage 16 (left column) and early-stage 17 (right column) wild-type, *Ptp4EPtp10D*, *Ptp4EPtp10D;rab5*^2^ and *Ptp4EPtp10D;vps45*^*JJ−2*^ embryos stained for the luminal protein Verm (grey). Scale bars, 10 μm. **b** Plots showing the average number of wild-type (*n* = 24), *Ptp4EPtp10D* (*n* = 66) and *Ptp4EPtp10D; rab5*^*2*^ (*n* = 59) mutant embryos with luminal Dextran-TR at 17.5 h after egg laying (1.5 h prior to the luminal clearance initiation in wild type). The premature luminal clearance phenotype of *Ptp4EPtp10D* is suppressed in *Ptp4EPtp10D;rab5*^*2*^ triple mutant embryos. **P < 0.001. **c** Plots depicting the average DT (Tr8) diameter in wild-type (*n* = 38), *Ptp4EPtp10D* (*n* = 45), *Ptp4EPtp10D;rab5*^*2*^*/+* (*n* = 28), *Ptp4EPtp10D;rab5*^*2*^ (*n* = 56), *Ptp4EPtp10D;vps45*^*JJ−2*^ (*n* = 29), *Ptp4EPtp10D;btl>Shi*^*RM*^ (*n* = 37), *Ptp4EPtp10D;rab11*^*j2D1*^ (*n* = 22), *Ptp4EPtp10D;btl>Rab11*^*S25N*^ (*n* = 47) and *Ptp4EPtp10D;btl>Rab11*^*Q70L*^ (*n* = 27) embryos. The DT diameter phenotype observed in *Ptp4EPtp10D* mutants is significantly restored in *Ptp4EPtp10D;rab5*^*2*^, *Ptp4EPtp10D;vps45*^*JJ−2*^ and *Ptp4EPtp10D;btl>Shi*^*RM*^ embryos. ***P < 0.001; **P < 0.005. Unpaired two tailed *t* tests were performed in relation to *Ptp4EPtp10D* mutant data set (**b**, **c**). Error bars show s.e.m.

To investigate a potential role for PTPs in apical endocytosis, we tested whether mutations reducing endosomal trafficking influence the defects of *Ptp4EPtp10D* mutants. The premature clearance of luminal Verm in *Ptp4EPtp10D* was suppressed by mutations in genes mediating early endosome fusion (Rab5 GTPase and the Rabenosyn-5/Rab5 interacting protein Vps45)^3,27–29^ (Fig. 2a). Further, Dextran-TR uptake assays showed that the precocious dextran internalization is reinstated by reduction of Rab5 levels in *Ptp4EPtp10D* (Fig. 2b). The expression of a dominant-negative form of *shibire*, the fly Dynamin, or the reduction of Rab5 or Vps45 in *Ptp4EPtp10D* mutants also suppressed the tube shape defects in *Ptp4EPtp10D* (Fig. 2a, c). Contrasting the effects of early endosomal trafficking mutations, interference or overexpression of the recycling endosomal GTPase Rab11 in *Ptp4EPtp10D* mutants had no effect on the *Ptp4EPtp10D* cystic tube phenotype (Fig. 2c). This suggests that PTPs preferentially downregulate early but not recycling endosomal trafficking. To investigate their roles in endocytosis, we first co-stained WT embryos expressing yellow fluorescent protein (YFP)-tagged endocytic Rabs (YRab5, YRab7, YRab11) or YRab1^30^, as a negative control with anti-Ptp10D and anti-green fluorescent protein (anti-GFP) antibodies. Apart from a pronounced apical surface staining, as in ref. ^7^, we detected that cytoplasmic Ptp10D-positive puncta co-stained with YRab5 and YRab7 and weaker with YRab11 or YRab1 (Supplementary Fig. 2a, b). This indicates that Ptp10D and likely also Ptp4E are found in endocytic compartments. To investigate endosomal traffic progression in the *Ptp4EPtp10D* mutant airways, we labelled ubiquitinated proteins destined for autophagosomal or lysosomal degradation with the dual GFP-mCherry reporter for Ref(2)P, the *Drosophila* p62 orthologue^31^. This reporter relies on the difference in fluorescence stability between mCherry and GFP in acidic pH^32^. We detected a relative increase in mCherry fluorescence and a concomitant decrease in GFP signal of *Ptp4EPtp10D* tracheal cells (Supplementary Fig. 2c) arguing for an enrichment of acidic endosomal compartments in *Ptp4EPtp10D* at the time of premature protein clearance. In addition, staining with an antibody that recognizes mono- and poly-ubiquitylated proteins (FK2) showed decreased accumulation of degradation-targeted proteins in the mutants compared to WT embryos (Supplementary Fig. 2d). These data further argue for a premature enhancement of endocytic trafficking towards degradation in *Ptp4EPtp10D* mutants. Taken together, the mutant analysis suggests that PTPs constrain the activation of the massive endocytic uptake of luminal material by modulating early endosomal trafficking. Moreover, reduction of early endocytosis in the mutants can rescue both premature clearance and tube shape defects suggesting a causative link between apical endocytosis and tube shape control.

### Btk29A antagonizes PTPs during luminal clearance

**Fig. 3:**
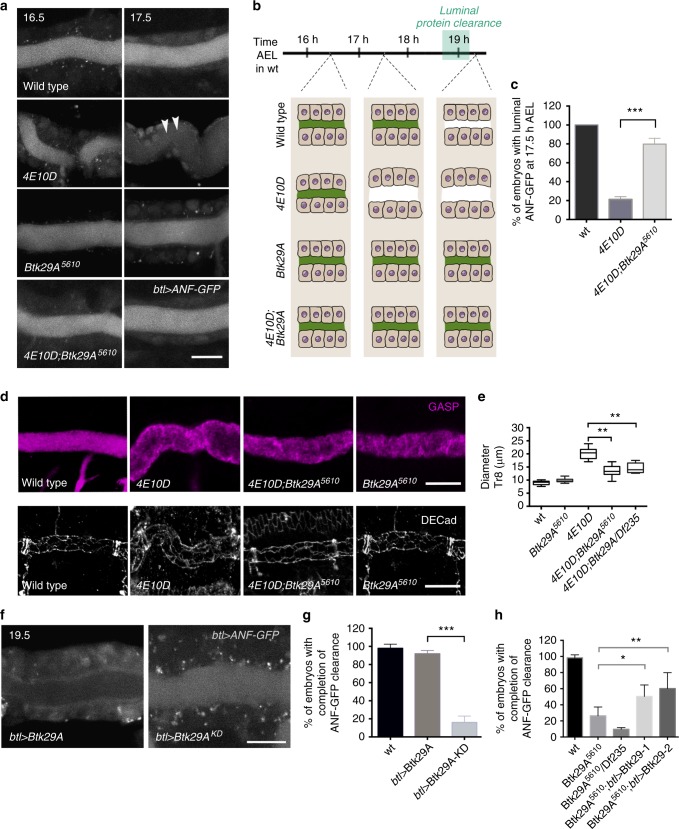
Btk29A mutations suppress the *Ptp4EPtp10D* phenotypes. **a** Confocal frames showing the dorsal trunk (DT) of living wild-type, *Ptp4EPtp10D, Btk29A*^*5610*^ and *Ptp4EPtp10D;Btk29A*^*5610*^ mutant embryos expressing *btl>ANF-GFP* (grey). The indicated time points represent hours after egg laying (AEL). The premature ANF-GFP luminal clearance in *Ptp4EPtp10D* (arrowheads) is repressed in *Ptp4EPtp10D;Btk29A*^*5610*^ triple mutant embryos. **b** Schematics of the luminal clearance in wild-type, *Ptp4EPtp10D, Btk29A*^*5610*^ and *Ptp4EPtp10D;Btk29A*^*5610*^ embryos. The time interval of luminal uptake in wild-type embryos is indicated (green box). **c** Graph showing the average number of wild-type (*n* = 39), *Ptp4EPtp10D* (*n* = 51) and *Ptp4EPtp10D;Btk29A*^*5610*^ (*n* = 65) embryos with luminal ANF-GFP at 17.5 h AEL (1.5 h prior to the luminal clearance initiation in wild-type). ***P < 0.0001 (unpaired two tailed *t* test). **d** Confocal images of the DT in wild-type, *Ptp4EPtp10D*, *Btk29A*^*5610*^ *and Ptp4EPtp10D;Btk29A*^*5610*^ mutant embryos (stage 17) stained for the luminal protein GASP (magenta in upper row panels) or the adherens junction protein DE-Cadherin (grey in lower row panels). **e** Plots depicting the average diameter of the DT (Tr8) in wild-type (*n* = 6), *Btk29A*^*5610*^ (*n* = 8), *Ptp4EPtp10D* (*n* = 7), *Ptp4EPtp10D;Btk29A*^*5610*^ (*n* = 8) and *Ptp4EPtp10D;Df235*/*Btk29A*^*5610*^ (*n* *=* 6) embryos (stage 17). The boxplot shows the median (internal line) and the range of the data is from 25th to 75th percentile. The bars present maxima and minima values. Unpaired two tailed *t* tests were performed in comparison to *Ptp4EPtp10D* data set. **P < 0.006. **f** Selected frames from live imaging of the tracheal DT of *btl>Btk29A* and *btl>Btk29A*^*KD*^ embryos expressing ANF-GFP (grey). **g** Plots showing the percentage of wild-type (*n* = 14), *btl>Btk29A* (Type 2 or long isoform) (*n* = 28) and *btl>Btk29A*^*KD*^ (*n* = 32) embryos that completed ANF-GFP luminal clearance. Unpaired two tailed *t* test was performed to compare *btl>Btk29A* and *btl>Btk29A*^*KD*^ data sets. ***P < 0.0001. **h** Plot showing the percentage of wild-type (*n* = 54), *Btk29A*^*5610*^ (*n* = 67), *Df235*/*Btk29A*^*5610*^ (*n* = 75), *Btk29A*^*5610*^;*btl>Btk29A-1* (type-1) (*n* = 58) and *Btk29A*^*5610*^;*btl>Btk29A-2* (type-2) (*n* = 72) embryos that complete luminal ANF-GFP clearance. *P < 0.01, **P < 0.001, (unpaired two tailed *t* tests). Error bars show s.e.m. Scale bars, 10 μm

To gain a mechanistic view on how PTPs control apical endocytosis and tube shape, we surveyed for potential interactors in airway maturation. We generated triple mutants, in which *Ptp4EPtp10D* mutants were combined with mutations in genes affecting RTK signalling components in *Drosophila*. In these triple mutant embryos, we assessed DT diameter at stage 17 and identified *Btk29A*^*5610*^ as a strong suppressor of the *Ptp4EPtp10D* tube shape phenotype (Supplementary Table 1). Btk29A contains SH2, SH3 and kinase domains while its longer isoform additionally contains a Btk homology domain and a pleckstrin homology (PH) domain (Supplementary Fig. 3a). Live imaging of *Ptp4EPtp10D;Btk29A*^*5610*^ embryos showed that reduction of Btk29A rescued the premature ANF-GFP clearance of *Ptp4EPtp10D* mutants (Fig. 3a–c). Antibody staining for DE-Cad and Gasp in *Ptp4EPtp10D;Btk29A*^*5610*^ triple mutants showed a restored DT shape compared to *Ptp4EPtp10D* (Fig. 3d, e). Since inactivation of Btk29A largely restores the lack of PTPs, we examined the phenotypes of *Btk29A* loss-of-function alleles in airway maturation. We used homozygous *Btk29A*^*5610*^ mutants^33^ and embryos trans-heterozygous for *Btk29A*^*5610*^ and a small chromosomal deletion (*Df235*) removing the *Btk29A* gene (*Df235/Btk29A*^*5610*^) to further diminish Btk29A protein levels (Supplementary Fig. 3h, i). Both *Btk29A*^*5610*^ and *Df235/Btk29A*^*5610*^ embryos develop a normal trachea system until stage 16 (Fig. 3d and Supplementary Fig. 3b, c) but fail to complete ANF-GFP (Fig. 3a, b) or Dextran-TR luminal clearance and gas filling (Supplementary Fig. 3d–g). Like *rab5*^*2*^ embryos at the launch of luminal clearance, 55% of *Btk29A*^*5610*^ mutants failed to initiate dextran internalization compared to WT embryos injected and imaged in parallel (Supplementary Fig. 3e). Similarly, tracheal overexpression of a Btk29A (type-2) version carrying the critical K554M mutation in the kinase domain (Btk29A^KD^) generated dominant-negative phenotypes in luminal and intracellular ANF-GFP accumulations (Fig. 3f, g). Re-expression of either Btk29A isoforms specifically in the mutant airways led to a significant restoration of ANF-GFP protein clearance but did not rescue the gas-filling defect (Fig. 3h and Supplementary Fig. 3g). These observations indicate that Btk29A kinase activity is required in the airways for initiation and completion of luminal material clearance. The failure to rescue the gas-filling phenotype can be explained by the requirement of Btk29A in posterior spiracle morphogenesis. These structures are also required for gas filling but do not express *btl*Gal4^34^. Alternatively, both isoforms may be required for gas filling in the airways after protein clearance. To further investigate the interplay of Btk29A and PTPs in endocytosis, we examined whether Btk29A overexpression affects tube shape and luminal protein uptake in PTP mutants. Although tracheal overexpression of Btk29A did not interfere with tube maturation in WT embryos (Fig. 3f, g and Supplementary Fig. 4a, b), its overexpression in *Ptp4E* single mutants, which lack tracheal phenotypes caused both DT shape defects and premature luminal protein clearance. In addition, overexpression of the Btk29A long isoform in *Ptp4EPtp10D* double mutant background further boosted DT expansion and premature clearance of ANF-GFP (Supplementary Fig. 4a, b). These results reveal that type III PTPs act as negative regulators of Btk29A during tube shape control and luminal clearance in *Drosophila* airways. This antagonism in dampening or promoting endocytosis prompted us to test the expression of each protein in mutants affecting the other. We did not detect any defects in the levels or localization of PTPs in *Btk29A* mutants or vice versa (Supplementary Fig. 3j–n), suggesting that they may antagonize by modifying each other’s activity or common downstream effectors.

### WASH is a critical Btk29A target

**Fig. 4:**
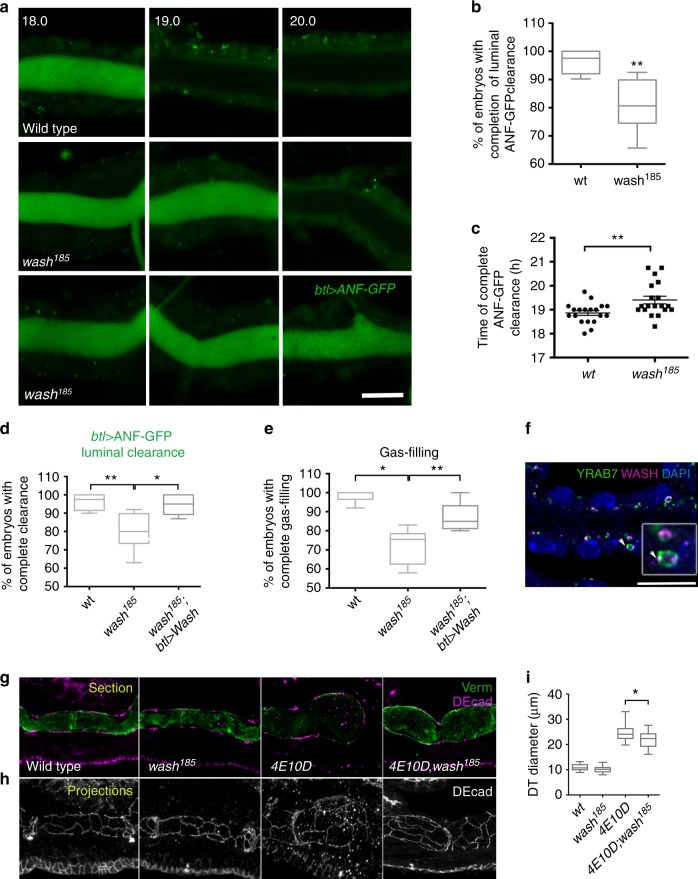
WASH (Wiskott–Aldrich syndrome protein and SCAR homologue) inactivation suppresses the *Ptp4EPt10D* tube shape phenotype. **a** Confocal frames showing the dorsal trunk (DT) of living wild-type and *wash*^*185*^ mutant embryos expressing *btl>ANF-GFP* (green). *wash*^*185*^ mutant embryos retain luminal ANF-GFP (lowest row) or delay clearance (middle row). Time points represent hours after egg laying (AEL). **b** Plots showing the percentage of wild-type (*n* = 48) and *wash*^*185*^ (*n* = 57) mutant embryos that complete ANF-GFP luminal clearance (20 h AEL). *P < 0.005 (unpaired two tailed *t* tests). **c** Plots depicting the average time (hours) of luminal ANF-GFP clearance completion in wild-type (*n* = 19) and *wash*^*185*^ (*n* = 19) mutant embryos. Error bars show s.e.m. **P < 0.001 (unpaired two tailed *t* test). **d**, **e** Plots depicting the percentage of wild-type (*n* = 27), *wash*^*185*^ (*n* = 32) and *wash*^*185*^*;btl>Wash* (*n* = 52) embryos completing the ANF-GFP luminal clearance (**d**) or gas filling (**e**). Re-expression of *Wash* specifically in the tracheal cells of *wash*^*185*^ mutants rescues the ANF-GFP luminal clearance and gas-filling defects. *P < 0.02; **P < 0.009 (unpaired two tailed *t* tests). **f** Confocal image depicting the DT of an embryo expressing endogenous YFP-tagged Rab7, stained with anti-WASH (magenta), anti-GFP (green) and DAPI (blue). Inset denotes zoomed view of a late endosome (arrowhead). Arrowheads indicate colocalization of WASH and Rab7. **g**, **h** Confocal images showing the tracheal DT of stage 17 wild-type, *wash*^*185*^*, Ptp4EPtp10D* and *Ptp4EPtp10D;wash*^*185*^ mutant embryos stained for Verm (green) and the AJ marker (DECad, magenta). Single sections (upper row) (**g**) and *Z*-stack maximum projections (lower row) (**h**). **i** Plots depicting the average DT diameter of wild-type (*n* = 12), *wash*^*185*^ (*n* = 14), *Ptp4EPtp10D* (*n* = 17) and *Ptp4EPtp10D;wash*^*185*^ embryos (*n* = 21). The DT diameter phenotype of *Ptp4EPtp10D* is significantly restored in the *Ptp4EPtp10D;wash*^*185*^ triple mutants. *P < 0.018 between *Ptp4EPtp10D* and *Ptp4EPtp10D;wash*^*185*^ embryos (unpaired two tailed *t* test). The boxplots show the median (internal line) and the range of the data is from 25th to 75th percentile. The bars present maxima and minima values. Scale bars, 10 μm

To identify potential downstream components, we focused on common regulators of actin polymerization and endocytosis. In *Drosophila*, WASH is not only required for cell motility and lysosomal neutralization in macrophages^35^ but also regulates the recycling of the luminal protein Serp in the airways^36,37^. Live imaging of *wash*^*185*^ mutants^38^ revealed normal secretion but defective clearance of the luminal protein ANF-GFP (Fig. 4a). Specifically, 20% of the analysed *wash*^*185*^ embryos failed to complete ANF-GFP clearance compared to WT (4.6%). The remaining mutants (80%) completed luminal clearance with a 35-min delay (Fig. 4a–c). *wash*^*185*^ embryos also accumulated luminal Dextran-TR during tube endocytic clearance and did not complete or partially completed gas filling (Supplementary Fig. 5a–c). Both the protein clearance and gas-filling defects of *wash*^*185*^ mutants were restored upon re-expression of *wash* in the airways (Fig. 4d, e). Staining with an anti-WASH antibody showed a punctate distribution in the airways and increased co-localization with the endosomal marker Rab7 during the period of luminal clearance (Fig. 4f and Supplementary Fig. 5d, e), resembling mammalian WASH localization on endosomes^16,17^. Similarly to *Ptp4EPtp10D;Btk29A*^*5610*^ mutants, *Ptp4EPtp10D;wash*^*185*^ triple mutants also showed a restoration of the *Ptp4EPtp10D* tube overexpansion phenotype, but we did not detect tube shape defects in the *wash*^*185*^ mutants (Fig. 4g–i). These results suggest that WASH would act downstream or in parallel to PTPs and Btk29A in airway maturation.

### Disruption of cortical F-actin promotes endocytosis

**Fig. 5:**
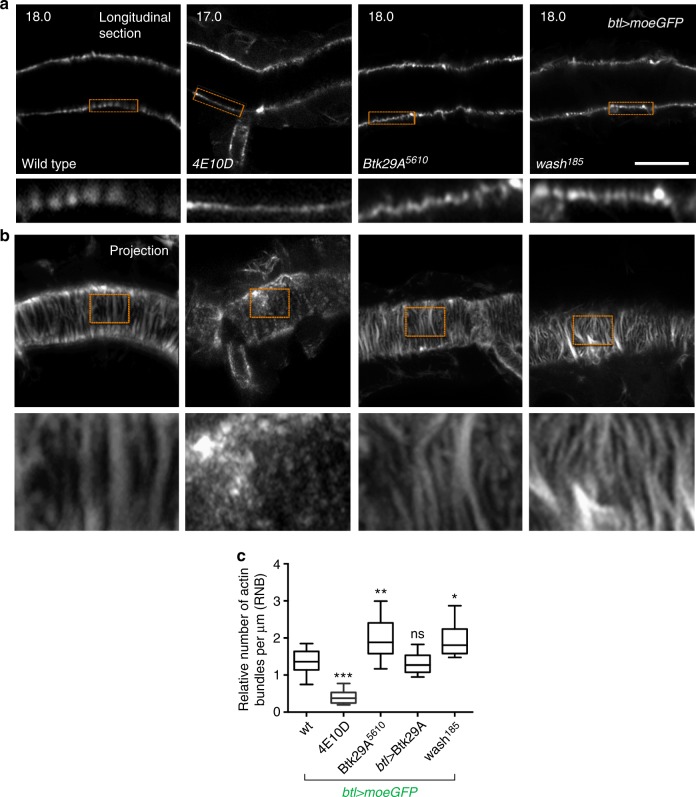
A new role of protein tyrosine phosphatases (PTPs) in actin cytoskeleton regulation. **a**, **b** Airyscan confocal images of the dorsal trunk of wild-type, *Ptp4EPtp10D, Btk29A*^*5610*^ and *wash*^*185*^ embryos expressing the actin reporter *btl>moeGFP* (grey). Images were acquired during luminal endocytosis, 18 h after egg laying (AEL) (for wild-type, *Btk29A*^*5610*^ and *wash*^*185*^) and 17 h AEL (for *Ptp4EPtp10D)*. Longitudinal sections (**a**) and *Z*-stack projections (**b**) are shown. Lower rows in **a** and **b** depict zoomed view of areas indicated by the rectangular frames. Scale bars, 10 μm. **c** Plots showing the relative number of actin bundles (>2 μm long) per μm (RNB) in wild type (*n* = 10), *Ptp4EPtp10D* (*n* = 8), *Btk29A*^*5610*^ (*n* = 14), *btl*>*Btk29A* (type-2) (*n* = 11) and *wash*^*185*^ (*n* = 14) embryos expressing *btl>moeGFP*. The boxplot shows the median (horizontal line) and the data range from 25th to 75th percentile. The bars denote maxima and minima values. *P < 0.01, **P < 0.001, ***P < 0.0001 indicates statistical significance in comparison to wild-type (unpaired two tailed *t* tests)

**Fig. 6:**
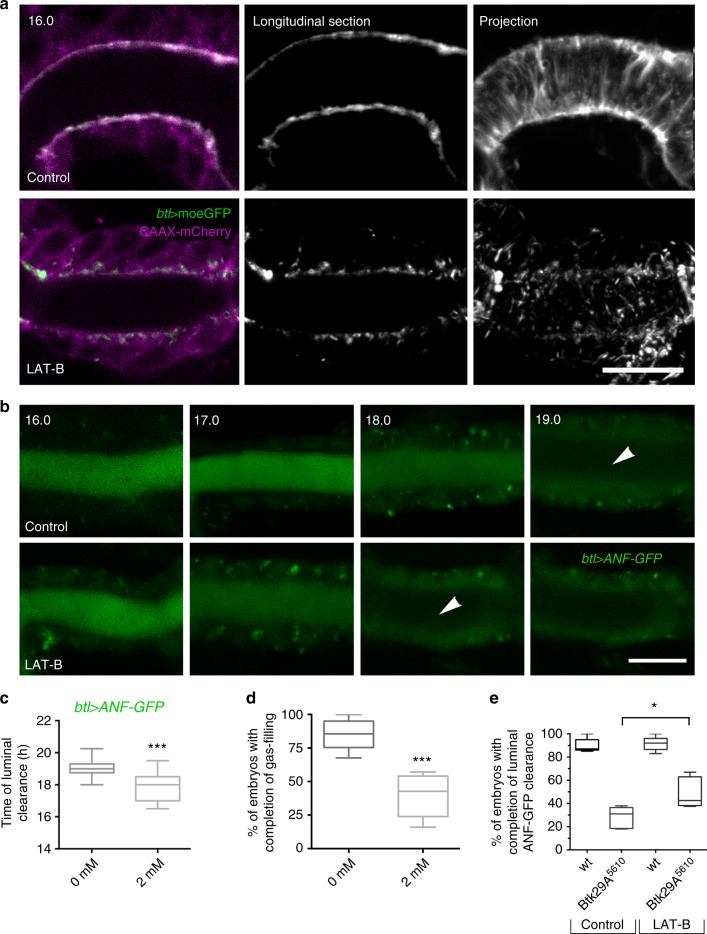
Chemical disruption of F-actin bundles induces apical endocytosis. **a** Airyscan confocal micrographs from live recordings showing the dorsal trunk (DT) of dimethyl sulfoxide (DMSO)-treated (control) or 2 mM (LAT-B)-treated wild-type embryos expressing the actin reporter *btl>moeGFP* (green and grey) and the membrane marker *btl>CAAX-mCherry* (magenta) in tracheal cells. Left and middle column show longitudinal sections. The right-most column depicts *Z*-stack projections. **b** Selected frames from confocal live imaging showing the DT of DMSO-treated (control) and 2 mM LAT-B-treated wild-type embryos expressing *btl>ANF-GFP* (green). LAT-B-treated embryos complete luminal *btl>ANF-GFP* clearance earlier than the controls (arrowheads). Arrowheads indicate the cleared tracheal lumen. Time points are hours after egg laying. **c**, **d** Plots showing the average time (hours) of luminal ANF-GFP clearance (**c**) or gas filling (**d**) in DMSO-treated (control) (*n* *=* 41) and LAT-B-treated (2 mM) wild-type embryos (*n* = 46). LAT-B-treated embryos clear luminal ANF-GFP significantly earlier. ***P < 0.001 denotes statistical significance (unpaired two tailed *t* tests). **e** Plots depicting the percentage of DMSO treated (control) wild-type (*n* = 50) and *Btk29A*^*5610*^ (*n* = 48) embryos as well as LAT-B-treated wild-type (*n* = 61) and *Btk29A*^*5610*^ embryos (*n* = 72) that complete luminal *btl>ANF-GFP* clearance. *P < 0.021 (unpaired two tailed *t* test). The boxplots show the median (internal line) and the range of the data is from 25th to 75th percentile. The bars present maxima and minima values. Scale bars, 10 μm

To investigate the link between the apical actin cytoskeleton and luminal endocytosis, we analysed the actin bundle organization in the mutants above expressing *btl>moeGFP* (Supplementary Fig. 1a) during luminal clearance. In *Btk29A*^*5610*^ and *wash*^*185*^ mutants, where luminal endocytosis initiation is disrupted or delayed, the apical actin bundle density was increased compared to WT. In *Ptp4EPtp10D*, where luminal endocytosis initiates earlier, bundles are established at 13 h AEL as in WT but become sparse and reduced to punctate accumulations at the time of premature clearance and thereafter (Fig. 5a–c and Supplementary Fig. 6a-c). These results show an inverse correlation of apical actin bundle organization with the activation of apical endocytosis in the different genotypes. To test a potential causative relationship between apical actin bundling and endocytosis, we first disrupted bundle formation in *btl>moeGFP* or *btl>LifeAct-GFP* embryos by timed injections of different concentrations of Latrunkulin B (LAT-B). Two-mM LAT-B injection disperses actin bundling but does not abolish apical F-actin accumulation (Fig. 6a and Supplementary Fig. 7a). In all, 40% of the treated embryos complete gas filling and hatch 2 h later (Fig. 6d). Importantly, the same LAT-B treatment in *btl*>ANF-GFP embryos induced premature luminal ANF-GFP clearance significantly earlier than in dimethyl sulfoxide (DMSO)-treated controls (Fig. 6b, c). Higher concentrations of LAT-B (10 mM) blocked luminal endocytosis (Supplementary Fig. 7b). This bimodal effect of systemic LAT-B on luminal endocytosis suppressing at high dose and accelerating at low dose suggests that LAT-B acts on two actin networks, one promoting endocytosis (endosomal F-actin) and the other suppressing endocytosis (cortical F-actin bundles). To test this scenario, we specifically targeted apical bundle formation by the transgenic expression of the dominant-negative form of the *Drosophila* formin DAAM (Dishevelled-associated activator of morphogenesis) (DAAM^C^)^12^. Under these conditions, the luminal secreted ANF-Cherry was cleared prematurely (Supplementary Fig. 7c–f). These data argue that cortical bundle disruption, either genetically or chemically, is sufficient to induce precocious massive luminal material uptake and that cortical actin bundles have a negative effect on actin-assisted endocytosis. *Btk29A*^*5610*^ mutants injected with 2 mM LAT-B lost their dense bundle arrangement and a larger fraction of them cleared the luminal marker ANF-GFP compared to *Btk29A*^*5610*^ embryos injected with DMSO (Fig. 6e). Altogether, we suggest that apical actin bundles keep F-actin-assisted luminal endocytosis at a steady-state low level and that a crucial function of Btk29A may be to antagonize the integrity of the actin bundles.

### Btk29A can phosphorylate WASH

**Fig. 7:**
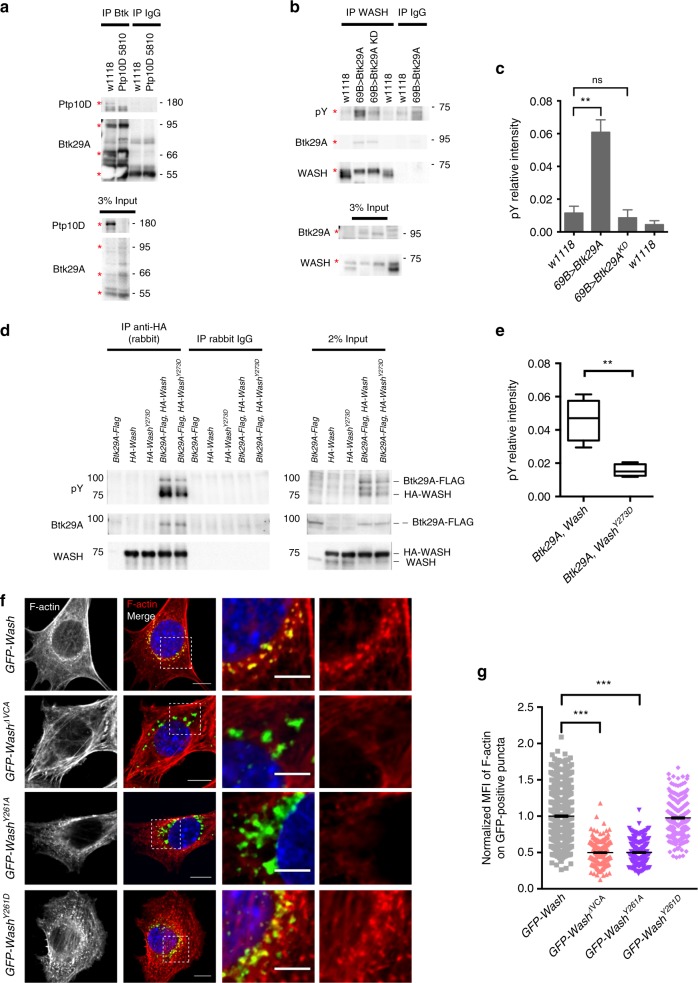
Btk29A forms complexes with Ptp10D and WASH (Wiskott–Aldrich syndrome protein and SCAR homologue). **a** Co-immunoprecipitates of Btk29A, using anti-Btk29A antibody or IgG (negative control), from protein lysates of wild-type (w1118) or *Ptp10D* mutant (#5810) embryos blotted with anti-Ptp10D and anti-Btk29A. Input (3%) is indicated. **b** Co-immunoprecipitates of WASH, with anti-WASH antibody or IgG (negative control) from protein lysates of wild-type, *69B>Btk29A*^*WT*^, *69B>Btk29A*^*KD*^ and wild-type embryos blotted with anti-pY, anti-Btk29A and anti-WASH. Input (3%) is indicated. **c** Bar graph depicting the relative pY levels of WASH immunoprecipitates shown in **b**. Error bars denotes s.e.m. (*n* = 3, independent immunoprecipitation (IP) experiments). **P < 0.004, ns not significant (*P* > 0.05) (unpaired two tailed *t* tests). **d** Co-immunoprecipitates of HA-tagged WASH protein from lysates of transfected S2 cells (using anti-HA antibody or rabbit IgG as a control), blotted with anti-pY, anti-Btk29A and anti-WASH. The cells were transfected with pAWF vector containing *Btk29A* cDNA and pAHW containing *Wash* or *Wash*^*Y273D*^ cDNA, as indicated. **e** Graph showing the relative pY intensity of the *Btk29A-Flag, HA-Wash* and *Btk29A-Flag, HA-Wash*^*Y273D*^ samples shown in **d**. **P < 0.0029 (unpaired two tailed *t* tests), *n* = 5 (independent IP experiments). The boxplots (**c**, **e**) show the median (horizontal line) and the data range from 25th to 75th percentile. Bars present maxima and minima values. **f** Confocal images showing staining for GFP (green), phalloidin (red) and Hoechst (blue) of WASH-knockout mouse embryonic fibroblasts (WASH^KO^ mouse embryonic fibroblasts (MEFs)) expressing *GFP-Wash*, *GFP-Wash*^*ΔVCA*^, *GFP-Wash*^*Y261A*^ and *GFP-Wash*^*Y261D*^. The two right columns display zoomed view of regions indicated by a rectangular frame in left columns. Representative images are shown. Scale bars, 10 μm on non-zoomed and 5 μm on zoomed images. **g** Scatter plots showing the normalized mean fluorescence intensity (MFI) of F-actin on GFP-positive puncta in *Wash* knockout MEFs cells expressing *GFP-Wash* (*n* = 616, puncta), *GFP-Wash*^*ΔVCA*^ (*n* = 249), *GFP-Wash*^*Y261A*^ (*n* = 199) and *GFP-Wash*^*Y261D*^ (*n* = 326). Values were normalized to the control (*GFP-Wash*). Two independent experiments from each condition are shown. Significance was calculated relative to the control (*GFP-Wash*) by unpaired two tailed *t* test; ***P < 0.0001

To explore the molecular interactions of Btk, WASH and PTPs, we first examined whether WASH, Btk29A and Ptp10D form complexes in the embryo. The anti-Btk29A antibody immunoprecipitated Ptp10D from WT embryonic lysates but not from *Ptp10D*^*5810*^ single mutants (Fig. 7a). Protein extracts from *Ptp4EPtp10D*, *Btk29A*^*5610*^ and WT embryos contain similar relative levels of WASH arguing that PTPs and Btk29A do not affect WASH expression but rather influence its activity (Supplementary Fig. 8a, b). Biochemical studies of the WASH-related WASP and N-WASP proteins showed that they are phosphorylated by Src kinase on Y^291^/Y^256 ^^39^. In the presence of GTP-bound Cdc42, this phosphorylation increases the basal activity of WASP towards the Arp2/3 complex and promotes actin polymerization^40^. Human, mouse and fly WASH are composed by three conserved regions, WHD1, WHD2 and VCA, and encompass a similar Y-containing motif in the corresponding position of their WASH homology domain 2 (WHD2) (Supplementary Fig. 9a, b). Immunoprecipitation (IP) of the endogenous WASH protein from embryo lysates showed low levels of pY, which increased significantly upon *Btk29A* but not by *Btk29A*^*KD*^ overexpression (Fig. 7b, c). In addition, we expressed HA-tagged *Wash* in S2 cells, immunoprecipitated with anti-HA antibodies and probed with anti-pY. We detected strong tyrosine phosphorylation of WASH when *Wash* was co-expressed with *Btk29A* but not with *Btk29A*^*KD*^ (Supplementary Fig. 8c, d). These data indicate that WASH can be phosphorylated and this phosphorylation requires Btk29A kinase activity. To examine whether WASH is a conserved substrate of Btk29A, we purified the three domains of the human WASH protein fused to glutathione *S*-transferase (GST)^18^ and examined their in vitro phosphorylation upon incubation with full-length human Btk kinase and P^32^-labelled γATP. In vitro, Btk phosphorylates WHD1 and WHD2 domains, but not VCA, which is devoid of the conserved tyrosine residues, or GST and bovine serum albumin (BSA) (negative controls) (Supplementary Fig. 8e).

We next focused on the conserved ANDLQ/MY motif of the WHD2 domain, which contains Y^273^ in *Drosophila*, Y^262^ in human WASH and Y^261^ in mouse WASH (Supplementary Fig. 9a, b). Co-expression of Btk29A-FLAG with HA-tagged, WT *Wash* or a mutated *Wash*^*Y273D*^ construct (phosphomimetic form) in S2 cells showed a stronger pY signal in HA-*Wash* compared to HA-*Wash*^*Y273D*^ upon IP with an anti-HA antibody (Fig. 7d, e). This suggested that Btk29A phosphorylates the conserved Y^273^ residue of *Drosophila* WASH. To establish the functional significance of the conserved ANDLQ/MY motif, we first used WASH-knockout mouse embryonic fibroblasts (WASH^KO^ mouse embryonic fibroblasts (MEFs)), where endosomes are devoid of actin patches^18^. In these cells, we re-expressed GFP-tagged versions of WT mouse *GFP-Wash*, *GFP-Wash*^*Y261A*^, *GFP-Wash*^*Y261D*^ or the inactive *GFP-Wash* ^*ΔVCA*^, which fails to stimulate Arp2/3 and assayed for F-actin intensities associated with GFP puncta. More F-actin accumulated on *GFP-WASH* and *GFP-Wash*^*Y261D*^ puncta, whereas *GFP-Wash*^*Y261A*^ or the *GFP-Wash*^*ΔVCA*^ mutants did not show increased F-actin accumulation (Fig. 7f, g). Similarly, *Drosophila wash*^*185*^ mutant embryos expressing the *Wash* but not a non-phoshorylatable *Wash* variant (*Wash*^*Y273F*^), restored the gas-filling defects (Supplementary Fig. 8f).

We also tested whether the *Wash*^*Y273D*^ interacts with the members of the SHRC by IPs in *Drosophila* S2 cells. Both HA-tagged Wash and HA-Wash^Y273D^ can immunoprecipitate the SHRC members strumpellin and CCDC53^19,20,22^. In a complementary experiment, IP with an anti-CCDC53 antibody precipitated similar levels of both HA- Wash and HA-Wash^Y273D^ (Supplementary Fig. 11a, b). These data indicate that the *Wash*^*Y261D*^ mutation does not disrupt the interactions of Wash with its regulatory complex. We conclude that WASH function in actin polymerization is regulated by this highly conserved tyrosine residue in both mammals and flies.

### WASH^Y273^ phosphorylation controls apical and endosomal actin

**Fig. 8:**
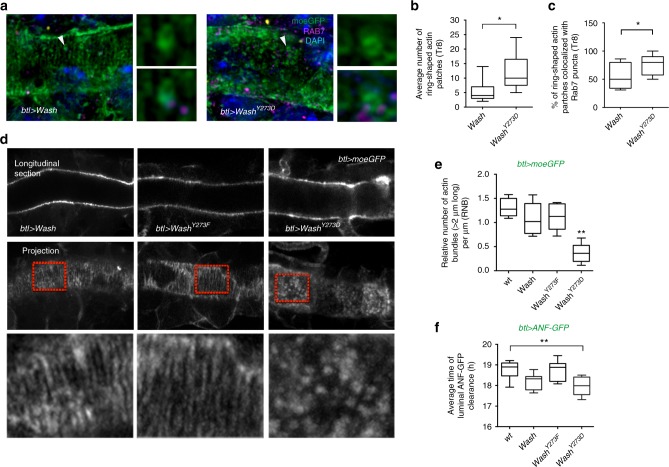
The WASH^Y273^ regulates actin organization and luminal clearance. **a** Airyscan confocal sections showing the dorsal trunk (DT) of *btl>Wash* or *btl>Wash*^*Y273D*^ embryos expressing the actin cytoskeleton reporter *moe-GFP* stained with anti-GFP (green), anti-Rab7 (magenta) and DAPI (blue). Images on the right of each confocal section depict zoomed views of Rab7-positive ring-shaped actin patches (arrowheads). **b**, **c** Plots showing the average number of ring-shaped actin patches in *btl>Wash (n* = 7*)* or *btl>Wash*^*Y273D*^ (*n* = 13) at 18 h after egg laying (AEL) (**b**), and the percentage of ring-shaped actin patches in *btl>Wash (n* = 8*)* or *btl>Wash*^*Y273D*^ (*n* = 14) colocalized with Rab7 puncta at 18 h AEL (**c**). Error bars show s.e.m. *P < 0.02 (unpaired two tailed *t* tests). **d** Airyscan confocal sections showing the DT of wild-type living embryos expressing *btl>Wash* or *btl>Wash*^*Y273F*^ or *btl>Wash*^*Y273D*^ and *moe-GFP* (grey) (18 h AEL). Images of the lowest row are zoomed areas of the cortical cytoskeleton indicated by the red rectangular frames. **e** Plots showing the relative number of actin bundles (>2 μm long) per μm (RNB) in wild-type (*n* = 14) and embryos expressing *btl>Wash* (*n* = 18) or *btl>Wash*^*Y273F*^ (*n* = 15) or *btl>Wash*^*Y273D*^ labelled by *btl>moeGFP* (*n* = 22). **P < 0.002 (unpaired two tailed *t* tests). **f** Plots showing average time (hours) of the luminal ANF-GFP clearance in wild-type (*n* = 66), *btl>Wash* (*n* = 38) or *btl>Wash*^*Y273F*^ (*n* = 39) or *btl>Wash*^*Y273D*^ (*n* = 41) embryos. **P < 0.001 compared to wild type (unpaired two tailed *t* tests). The boxplots (**b**, **c**, **e**, **f**) show the median (horizontal line) and the range from 25th to 75th percentile. The bars depict maximum and minimum values. Data collected from 5–7 independent experiments

To further test the functional significance of the Y^273^ phosphorylation in polarized epithelial tissues, we compared *Drosophila* embryos overexpressing WT *Wash*, *Wash*^*Y273F*^ or the *Wash*^*Y273D*^ variants in the airways (Supplementary Fig. 9b). We tested their effects on the apical cytoskeleton organization and on protein clearance initiation by imaging embryos co-expressing the *moe*-GFP or the ANF-GFP reporters. Airway-specific overexpression of *Wash* and *Wash*^*Y273F*^ mildly interfered with the organization of the apical bundles, but the phosphomimetic *Wash*^*Y273D*^ dispersed the long apical filaments and instead induced punctate and circular actin accumulations in the airway cells (Fig. 8a–e). These structures colocalized with Rab7 suggesting that *Wash*^*Y273D*^ induces endosomal actin polymerization and destabilizes cortical actin bundles (Fig. 8a–c). Further, overexpression of *Wash*^*Y273D*^ but not *Wash*^*Y273F*^ or *Wash* induced luminal clearance of ANF-GFP significantly earlier (Fig. 8f). The dominant *Wash*^*Y273D*^ phenotypes on localized actin polymerization and luminal protein clearance suggest that WASH activity is tightly regulated by Y^273^ phosphorylation. WASH activates the Arp2/3 complex to induce endosomal actin polymerization^16,17,41^ and we asked whether Y^273^ phosphorylation impinges on the Arp2/3 complex activity. The *Drosophila* Arp2/3 complex includes Arpc1 and Arp3. *Arpc1* mutants fail to clear luminal ANF-GFP at the end of embryogenesis suggesting that Arp2/3-mediated actin polymerization is indeed required for luminal endocytosis (Supplementary Fig. 10e). We then analysed the effect of *Wash*^*Y273D*^ overexpression in the background of loss-of-function mutations (*Arpc1*^*Q25sd*^, *Arp3*^*3640*^) affecting Arpc1 and Arp3^42^. *Wash*^*Y273D*^-induced punctate actin polymerization and premature luminal clearance of ANF-GFP was significantly suppressed in the *Arpc1* or *Arp3* mutants (Supplementary Fig. 9c–e) suggesting that the Wash^Y273^ modification influences the activity of the Arp2/3 complex. In line with the proposed regulatory model, *Ptp4EPtp10D;Arpc1* triple mutants showed milder tube shape and premature luminal clearance defects compared to the *Ptp4EPtp10D* mutants (Supplementary Fig. 10a–d). This genetic analysis suggests that PTPs control the activity of Arp2/3 through or in parallel to *Wash*.

## Discussion

**Fig. 9:**
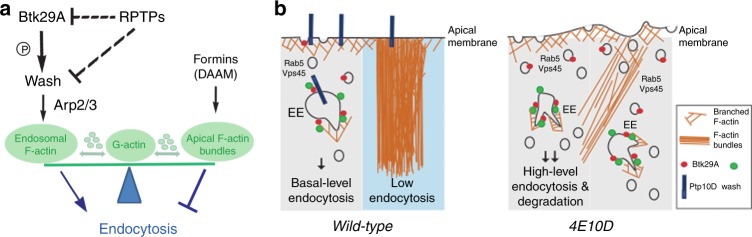
Receptor protein tyrosine phosphatase (PTP) and Btk regulation of F-actin network integrities. **a** Schematic representation of the interplay between PTPs, Btk29A and WASH (Wiskott–Aldrich syndrome protein and SCAR homologue) in endocytosis. Btk29A promotes WASH activation by tyrosine phosphorylation while PTPs directly or indirectly antagonizes WASH. Active WASH induces the formation of endosomal actin patches via the Arp2/3 complex and promotes endocytosis. By contrast, DAAM and formins generate apical/cortical actin bundles^12^ and maintain steady-state levels of endocytosis. The model proposes a direct competition between the WASH-Arp2/3 and formin-mediated polymerization modes for a globular actin pool (G-actin). **b** Cartoons showing the function of PTPs/Btk29A/WASH and actin bundles during luminal protein clearance in wild-type and *Ptp4EPtp10D*. EE early endosome

The apical cytoskeleton is the focus of continuous regulation during *Drosophila* airway maturation. Src kinases control cell shape changes and cell elongation by promoting the activity of formins^43,44^. Similarly, Btk29A is involved in the formation of DAAM-formin-dependent actin cables that prefigure the formation of spiral taenidial ridges ensuring tube integrity in larval airways^12,23,25^. Our work reveals a new function of Btk29A in regulating endosomal actin polymerization, endocytosis and the luminal airway clearance (Fig. 9a, b). A critical substrate of Btk29A is WASH, which activates the Arp2/3 complex on endosomal and lysosomal membranes and thereby controls vesicle morphology, scission and various endosomal routes, leading to degradation, recycling and retrograde transport^45^. In *Drosophila* and mammalian cells, assembly into the conserved SHRC regulates the constitutive activity of WASH towards the Arp2/3 complex^17,22^. K-63 ubiquitination of WASH on K220 releases the autoinhibitory conformation and promotes endosome to Golgi retrograde transport^46,47^. This effect is mediated by the FAM21 adaptor, which links the WASH complex to the retromer^22,48^. K220 is not conserved in invertebrates implying the presence of additional posttranslational modifications to accommodate WASH regulation. Our identification of the conserved Y^273^ motif in fly, human and mouse WASH and the effects of the WASH^Y273D^ mutant on actin organization and protein clearance suggests that, in addition to Rho activation, phosphorylation is a general mechanism regulating WASH-mediated actin polymerization and endosomal transport.

Earlier studies in *Drosophila* epithelial tissues and mammalian cells showed that Ptp4E and Ptp10D dampen RTK signalling^5,9,49^. Btk29A, on the other hand, is a positive RTK effector^13,15^. The induced phosphorylation of WASH by Btk29A overexpression and the suppression of *Ptp4EPtp10D* phenotypes by WASH reduction suggest an antagonistic relationship of PTPs and Btk29A directly on WASH phosphorylation. Alternatively, PTPs may dampen RTK signalling and thereby Btk29A recruitment and subsequent WASH phosphorylation on endosomes. In mammalian cells, WASH is required for endosome fission and the fast transport of EGF to late endosomes^16,41^. RTK signalling proceeds in endosomal compartments and recent work indicates that the number of endosomes with activated EGFR determines the signalling strength of the receptor. It is tempting to speculate that balancing endosomal WASH phosphorylation by EGFR effectors may be part of the proposed analogue-to-digital conversion that determines the signalling output by increasing or decreasing the number of endosomes carrying activated receptors^50^. Our identification of a conserved WASH regulatory mechanism provides an entry point into the interplay of endosomal actin polymerization and signalling during development and disease.

The genetic analysis of endocytosis regulation suggests an intriguing, antagonistic relationship between endosomal and apical bundled actin in epithelial tissues. In *Ptp4EPtp10D* mutants, the actin bundles are reduced and endocytic luminal clearance commence earlier, leading to premature luminal protein degradation. The irregular tube shapes in these mutants likely reflect a role of the perpendicularly oriented apical bundles in maintaining the organ shape during the early tube expansion interval. On the other hand, in *Btk29A* mutant apical bundles increase and apical endocytosis is reduced. These inverse correlations suggest that PTPs and Btk29A activities may either regulate both endosomal and actin bundle networks independently or that the two modes of assembly directly compete for a limiting globular actin pool^51^. The concomitant increase in endosomal patches and the reduction of apical bundles in embryos overexpressing WASH^Y273D^ may favour the view of a direct competition between the WASH-Arp2/3 and formin-mediated polymerization modes for a limited globular actin pool (Fig. 9a, b). However, it does not exclude that two actin networks regulate each other by more elaborate mechanisms and that additional phosphorylated substrates other than WASH may independently balance the growth of the two actin networks.

## Methods

### *Drosophila* strains

Mutants and transgenic fly strains used were: *Ptp4EPtp10D or 4E10D*^8^, *Btk29A*^5610^ (#102398, Kyoto Stock Center; KSC), *rab5*^2,27^, *vps45*^*JJ−2* 29^*, wash*^*185*^ (or *wash*^*Δ185*^, #28285,Bloomington Stock Center, BSC), *Df235* (#9710, BSC), *Arpc1*^*Q25sd*^ (#9137, BSC), *Arpc1*^*CH60*^ (#3585, BSC), *Arp3*^*3640*^ (#17149, BSC), *btl*>GAL4^52^, UAS-*Ptp4E*^8^, UAS-ANF-GFP (EMD)^3^, DE-Cad-GFP (Fluorescent knock-in allele of *DE-Cadherin*^53^), Viking (Vkg)-GFP^54^, UAS-*EGFR*^*CA*^ *(#1564*, BSC*)*, UAS-*EGFR*^*DN*^ (#5364, BSC), UAS-*FGFR*^*λTOP*^^55^, *Src64B*^56^, *Src42A*^*E*1^ (#6408, BSC), *egfr*^*k05115*^ (#10385, BSC), *Btk29A-1* (type-1, #109095, KSC), UAS-*Btk29A-2* (type-2, #109093, KSC), UAS-*Rab5*^*DN*27^, UAS-*shi*^*RM*^ ^57^, UAS-YFP-*Rab11*^*S25N*^ (#23261, BSC), *rab11*^*j2D1*^ (#12148, BSC), UAS-*YFP-Rab11*^*Q70L*^ (#9791, BSC), UAS-*Rab11-GFP* (#8506, BSC), fluorescent knock-in Rabs (YRab1, YRab5, YRab7, YRab11^30^), UAS-*moe*-GFP^58^, UAS-*CAAX-m-cherry* (#59021, BSC), UAS-*myr-mRFP*^1^ (#7118, BSC), UAS-*Ref(2)P-GFP-mCherry*^32^, UAS-*LifeAct-GFP* (#57326, BSC), UAS-*DAAM-*^*WT−FLAG*^, *UAS-DAAM*^*C*^ ^12^. *w*^*1118*^ (#5905, BSC) was used as the WT strain. Flies were raised at 25 °C.

### Transgenic flies

Complementary DNA (cDNA) encoding for *Drosophila* Ptp10D (clone RE52018) was cloned into the pJFRC-MUH or pJFRC28 (JFRC28–10XUAS-IVS-GFP-p10) vector (Addgene, plasmid 26213 and plasmid 36431, respectively) using NotI and KpnI restriction sites for the development of UAS*-Ptp10D* and UAS*-Ptp10D-GFP*, respectively. cDNA encoding for *Drosophila* Btk29A type-2 isoform (clone LD16208) was cloned into pJFRC-MUH or pJFRC28 vector using NotI and KpnI restriction sites for the development of UAS*-Btk29A* and UAS*-Btk29A-GFP*, respectively. The catalytically inactive Btk29A^KD^ (or Btk29A^K554M^) was generated by standard PCR-based site-directed mutagenesis (AAG to ATG) using Phusion High Fidelity DNA polymerase (New England Biolabs, M0530), followed by sub-cloning into pJFRC-MUH or pJFRC28 vector using NotI and KpnI restriction sites, for the development of UAS*-Btk29A*^*KD*^ and UAS*-Btk29A*^*KD*^*-GFP*, respectively. cDNA encoding for *Drosophila* WASH (clone RH66493) was cloned into pJFRC-MUH vector for the development of UAS*-Wash*. The mutated WASH variants *Wash*^*Y273F*^ and *Wash*^*Y273D*^ were generated by standard PCR-based site-directed mutagenesis (TAT to TTC or GAT, respectively) using Phusion High Fidelity DNA polymerase, followed by sub-cloning into pJFRC-MUH for the development of UAS*-Wash*^*Y273F*^ and UAS*-Wash*^*Y273D*^. The sequences of primers used for cloning are provided in Supplementary Table 2. The above constructs were used for the generation of PhiC31 integrase-mediated transgenesis on the second or third chromosome according to standard protocols.

### S2 cells

*Drosophila* S2 cells (Invitrogen, Carlsbad, CA) were cultured at 25 °C in Schneider’s *Drosophila* medium (GIBCO-Invitrogen, Carlsbad, CA), supplemented with 10% heat inactivated foetal calf serum, 2 mM L-glutamine, 100 U/ml penicillin and 100 mg/ml streptomycin. Flag-tagged Btk29A and HA-tagged WASH variants were developed using the Gateway system (ThermoFisher Scientific). *Btk29A* and *Btk29A*^*KD*^ cDNAs were introduced into the pENTR/D-TOPO (ThermoFisher Scientific #K240020) and subcloned into *pAWF* destination vector (DGRC #1112) and *wash*, *wash*^*Y273F*^ and *wash*^*Y273D*^ into the *pAHW* (DGRC #1095) using the Gateway LR Clonase II (ThermoFisher Scientific #11791020).

For the expression of Btk29A, Btk29A^KD^ and WASH variants, S2 cells were cultured on six-well plates for 18–24 h. They were transiently transfected with *pAWF-Btk29A*, *pAWF-Btk29A*^*KD*^, *pAHW-wash* or *pAHW-wash*^*Y273D*^ vectors and combinations. The cells were harvested 42 h post transfection. The sequences of primers used for cloning are provided in Supplementary Table 2.

### Co-IP and western blot analysis

For western blot analysis, *Drosophila* embryos were collected 12–20 h AEL and lysed in 20 μl of lysis buffer containing 50 mM HEPES (PH 7.6), 1 mM MgCl_2_, 1 mM EGTA, 50 mM KCl, 1% NP40, Protease inhibitor cocktail tablets (Roche #11697498001) and Phosphatase inhibitor cocktail 2 (Sigma Aldrich #P5726). The lysates were centrifuged at maximum speed (30,060 × *g*) for 10 min at 4 °C. Protein loading buffer (50 mM Tris/HCl, pH 6.8, 2% sodium dodecyl sulfate (SDS), 5% glycerol, 0.002% bromophenol blue) was added to the supernatant and samples were analysed by SDS-polyacrylamide gel electrophoresis (PAGE) and immunoblotting according to standard protocols, using the ChemiDoc XRS + system (BioRad), after application of the SuperSignal West Femto Maximum Sensitivity Substrate (ThermoFisher Scientific, 34096) or the Infrared Odyssey System (Li-Cor Biosciences).

For IP in embryos, 400 μl of 12–20 h AEL embryos were collected and lysed in 500 μl of lysis buffer (as above with the addition of 100 mM NaCl). The lysates were centrifuged at 1500 × *g* for 10 min and at maximum speed for 1 min at 4 °C to remove the debris. Part of the supernatant (30 μl) was kept as input, while the rest was divided into two equal parts and incubated either with antibodies or IgG control for 4 h. BSA (2 mg/ml) blocked Protein G sepharose beads (30 μl) (GE Healthcare, #10157795) were added to lysate-antibody/IgG mix and incubated for 1 h at 4 °C with rotating. The beads were collected and washed three times for 10 min with washing buffer (lysis buffer without 1% NP40 and with 200 mM NaCl). Proteins bound to the beads were eluted in protein loading buffer and analysed by SDS-PAGE and immunoblotting. The chemiluminescent signal was detected using the ChemiDoc XRS+ system (BioRad) after application of the SuperSignal West Femto Maximum Sensitivity Substrate (ThermoFisher Scientific, 34096).

For IP experiments from S2 lysates, S2 cells from two wells (of a six-well plate) were pulled together and lysed in 600 μl of lysis buffer (described above) for 30 min at 4 °C. Lysates were centrifuged at 1500 × *g* for 10 min at 4 °C. Thirty microlitres of the supernatant was kept as input and the rest was added to 25 μl Dynabeads (Thermo Scientific) coupled to the antibody of interest or 25 μl anti-HA affinity matrix (Sigma, #11815016001). The mix was incubated for 40 min at room temperature with mild shaking. The beads were washed according to the manufacturer’s protocol and the proteins were eluted in protein loading buffer and analysed by SDS-PAGE and immunoblotting.

The following primary antibodies were used at the indicated dilutions: rabbit anti-HA (1:2000, Abcam, ab9110), rabbit anti-FLAG (1:2000, Abcam, ab1162), mouse anti-FLAG M2 (1:3000, Sigma, F3165), rat anti-Btk29A (1:1000, (Tsikala et al.^34^)), mouse anti-Ptp10D (mixture of 8B22F5 and 45E10 antibodies used in 1:50 each, DSHB), mouse anti-WASH (1:10, P3H3, DSHB), rabbit anti-α-tubulin (1:2000, Cell Signaling, 11H10), rabbit anti-PhosphoTyrosine (P-Tyr-1000, 1:2000, Cell Signaling Technology, 8954), mouse polyclonal anti-Strumpellin (1:1000)^20^, mouse polyclonal anti-CCDC53 (1:1000)^20^ and mouse polyclonal anti-FAM21 (1:1000)^20^.

### Quantification of western blots

For the IP analysis, optical intensities of the protein bands of interest were measured using the ImageJ software. The actual signal intensity values of pY, WASH (in the IP) and Btk29A (Input) were estimated after subtraction of the background intensity of each lane. The pY intensity (phosphorylated WASH) of each sample was divided to the respective WASH signal intensity (total amount of immunoprecipitated WASH protein). Since variations in the amount of Btk29A kinase could affect the phosphorylation levels, the values were divided by the corresponding Btk29A intensity (estimate of the overall Btk29A kinase). For the western blot analysis, the actual signal intensity of each band of interest was estimated after subtraction of the background. The values were then divided by the corresponding intensities of the loading control (α-tubulin).

### In vitro kinase assay

The three domains of the human WASH protein were GST-purified^18^ according to standard protocols. For the in vitro protein phosphorylation assay, 100 ng of recombinant full-length human Btk kinase (Promega, V2941) was combined with 2 μg of GST, GST-WHD1, GST-WHD2 or GST-VCA in a kinase buffer solution (40 mM Tris/HCl pH 7.4, 20 mM MgCl_2_, 0.1 mg/ml BSA, 50 μM DTT, 2 mM MnCl_2_) supplemented with 100 μM ATP and 10 μCi [γ-^32^P] ATP (6000 Ci/mmol) EasyTide (PerkinElmer). The mixture was incubated at 30 °C for 30 min and the reaction was stopped by adding Laemmli sample buffer. The samples were boiled at 100 °C for 10 min and loaded on an SDS-PAGE. The gel was stained with Coomassie blue, dried and autoradiographed.

### Immunostaining

Embryos were bleached, dechorionated and fixed for 20 min in 4% formaldehyde saturated heptane as described in ref. ^59^. Embryos expressing *moeGFP* were dechorionated and devitellinized by hand. The following antibodies were used: mouse anti-Ptp10D (1:10, 8B22F5, Developmental Studies Hybridoma Bank, DSHB), rat anti-DE-Cad (1:50, DSHB), mouse anti-Rab7 (1:100, DSHB), mouse anti-Crb (1:10, Cq4 DSHB), mouse anti-Coracle (1:100, C615.16 DSHB), rabbit anti-GFP (1:400, A11122, Thermo Scientific), chicken anti-GFP (1:400, abl3970, Abcam), gp anti-Verm^60^, gp anti-Gasp^61^, rabbit anti-Uif^62^, gp anti-Uif^63^, mouse anti-WASH (1:5, P3H3, DSHB), and mouse anti-FasIII (1:10, 7G10 DSHB). Secondary antibodies conjugated to Cy2, Cy3 or Cy5 or Alexa Fluor-488 and -568 (Jackson Immunochemicals) were used and diluted as recommended by the manufacturer.

### Dextran and drug injections

For dye-permeability assays, 10 kDa Dextran-TR (ThermoFisher Scientific) was injected into late-stage 16 embryos (after the formation and maturation of SJ) as described in ref. ^64^. For endocytotic assays, we injected 10 kDa Dextran into the haemocoel of stage 13–14 embryos, as described^3^. Dextran and drugs were injected laterally or posteriorly in embryos mounted on a coverslip with heptane/glue and covered with halocarbon oil (#700) (Sigma). LAT-B was diluted in 6% DMSO and 94% H_2_O and different concentrations (0.5, 1, 2, 10, 50 mM) were injected, yielding approximately a dilution of 50-fold in the embryo^65^. All drug injections were performed in late-stage 16 (15.5 h AEL) dechorionated embryos using a microinjection system (FemtoJet, Eppendorf) coupled to an inverted fluorescence microscope (Cell Observer, Zeiss).

### Live imaging

Dechorionated embryos were mounted as described in ref. ^3^. For widefield imaging, embryos were imaged with a CCD camera (AxioCam 702, Carl Zeiss) attached to an AxioImagerZ1 or Z2 (Carl Zeiss) microscope by using either a ×20/0.75 NA Plan-Apochromat or a ×63/1.3 NA C-Apochromat objective (Carl Zeiss). *Z*-stacks with a step size of 0.5–1.9 μm were taken every 5–10 min over a 8–10-h period. The embryos used for measuring the time of luminal ANF-GFP or GASP-GFP or Dextran-TR clearance were collected for 15 min, dechorionated, and imaged by widefield microscopy. For conventional confocal live imaging, embryos were imaged with a laser-scanning confocal microscope (LSM 510 META or LSM780, Carl Zeiss) using a ×63/1.2 NA C-Apochromat water-immersion objective. Individual *Z*-stacks with a step size of 0.5–1.0 μm were taken every 6 min over a 3–8-h period. For high-resolution confocal imaging, an airyscan-equipped confocal microscope system (Zeiss LSM 800, Carl Zeiss) was used. *Z*-stacks (0.16–0.2-μm step size) were taken every 15 min over a 3–4-h period with a ×63/1.4 NA oil immersion objective. Raw data were processed with the airyscan processing tool available on the Zen Black software version 2.3 (Carl Zeiss). Images were converted to tiff format using the ImageJ/Fiji software.

### Morphometric analysis

The tube diameter and length measurements were conducted in embryos expressing *btl-mRFP-moe* and *btl>α-Cat-GFP* or stained for the apical markers DE-Cad or Uif. The diameter was measured at six separate positions within a tracheal metamere in each sample to reduce the tube-tapering effect. Tube length was measured by tracing the apical labelling between fusion cells. To measure the relative number of actin bundles (RNB), horizontal lines along the tube axis (1 μm apart) were drawn using the Zen software version 2.3. Actin bundles crossing at least the three lines were considered as positive (Supplementary Fig. 6c) and calculated as RNB per μm. Data sets were collected in text format and analysed using the Graph Pad Prism 6.0h. Intracellular Dextran-TR puncta were quantified by the particle analyser of ImageJ/Fiji software.

### MEF lines

WASH-knockout MEFs stably reconstituted with *HA.GFP-mWASH* WT and ΔVCA were previously described^18^. *WASH*^*Y261A*^ and *WASH*^*Y261D*^ mutant constructs were generated from *HA.GFP-mWASH WT* using the QuikChange II Site-Directed Mutagenesis Kit (Agilent, Santa Clara, CA) according to the manufacturer’s instructions. Retrovirus were produced using the GP2 packaging line from Clontech (Mountain View, CA). Transduced cells were selected in 1 μg/ml puromycin as previously described^18^.

### Immunofluorescence (IF) staining in MEF cell lines

MEFs were grown directly on coverslips at 37 °C. The coverslips were then prepared for IF as described^17^. Briefly, cells were fixed in ice-cold fixative (4% paraformaldehyde in phosphate-buffered saline [PBS]) for 18 min at room temperature in the dark, followed by permeabilization for 3 min with 0.15% Surfact-Amps X-100 (28314, ThermoFisher Scientific, Rockford, IL) in PBS. After three washes in PBS, cells were blocked in IF buffer (Tris-buffered saline plus human serum cocktail) for 30 min. Cells were then incubated with anti-GFP primary antibody (A-11120, ThermoFisher Scientific, Rockford, IL) in IF buffer overnight at 4 °C in a humidified chamber. After three washes in PBS, cells were incubated with secondary donkey anti-mouse IgG (H+L), Alexa Fluor 488 antibody and rhodamine–phalloidin (R415) (Life Technologies, Grand Island, NY) in IF buffer for 1 h at room temperature in a humidified chamber. After four washes in PBS, coverslips were stained with Hoechst 33342 (H3570) for nuclei, rinsed in water and affixed to slides with SlowFade Anti-fade reagent (Life Technologies, Grand Island, NY). Images were obtained using LSM-710 (×63/1.4 oil immersion objective lens; Carl Zeiss) confocal microscope.

### F-actin mean fluorescent intensity (MFI) and colocalization quantification

For the quantification of F-actin MFI on GFP-Wash-positive puncta in MEFs, 2 μm^2^ squares around the GFP-puncta were defined as the regions of interests (ROIs). MFI of F-actin within these ROIs was measured in Fiji. Data were normalized by dividing the fluorescence intensity value of each pixel by the average fluorescence intensity observed in the ROI of control cells. For the colocalization analysis in airways, Ptp10D-Cy3 or WASH-Cy3 puncta were used as ROIs. Colocalization of these Cy3 ROIs with the YFP-Rabs signals was quantified as the Pearson’s correlation coefficient *r*^66^ using Costes method^67^ for automatic thresholds setting. Image processing, ROI determination and *r* calculation were carried out with the ZEN software (blue edition, Carl Zeiss) or the Fiji plug-in Coloc2 (National Institutes of Health: http://rsb.info.nih.gov/ij/). For quantifying the number of colocalized actin patches with Rab7, ring-shaped GFP patches (green) with >25% overlapping with Rab7-Cy3 (magenta) were counted, using the ZEN software (blue edition, Carl Zeiss). This number was divided by the total number of ring-shaped GFP-patches to determine the percentage of their colocalization with Rab7 puncta in each embryo.

### Statistical analysis

Statistical analysis was carried out using two-tailed *t* test for unpaired variables unless indicated. The type of statistical test, *n* values and *P* values are all listed in the figure legends. All statistical analyses were performed using Graph Pad Prism 6.0h. For all the experiments, the number of biological replicates is indicated in the figure legends.

### Reporting summary

Further information on research design is available in the Nature Research Reporting Summary linked to this article.

## Supplementary information

SUPPLEMENTARY INFORMATION

Peer Review

Reporting Summary

Source Data

## Data Availability

The source data underlying Figs. 1d, e, f, h, 2b, c, 3c, e, g, h, 4b–e, h, 5b, 6c–e, 7c, e, g, 8b, c, e, f and Supplementary Figs. 1b, 2b, 3c, g, i, l, n, 4b, 5b, 7b, d, f, 8b, f, 9d, e, 10b, d, e are provided as Source Data file. All reagents and further experimental data are available from the corresponding author upon reasonable request.
