# Supplementary material for: WASH phosphorylation balances endosomal versus cortical actin network integrities during epithelial morphogenesis
Source: Nat Commun. 2019 May 16;10:2193. doi: 10.1038/s41467-019-10229-6 (PMC6522504; doi:10.1038/s41467-019-10229-6)
Supplement: Supplementary file 1 — SUPPLEMENTARY INFORMATION [file 41467_2019_10229_MOESM1_ESM.pdf]

Supplementary Information

WASH phosphorylation balances endosomal versus cortical actin network integrities during epithelial morphogenesis

Tsarouhas *et al*., 2019

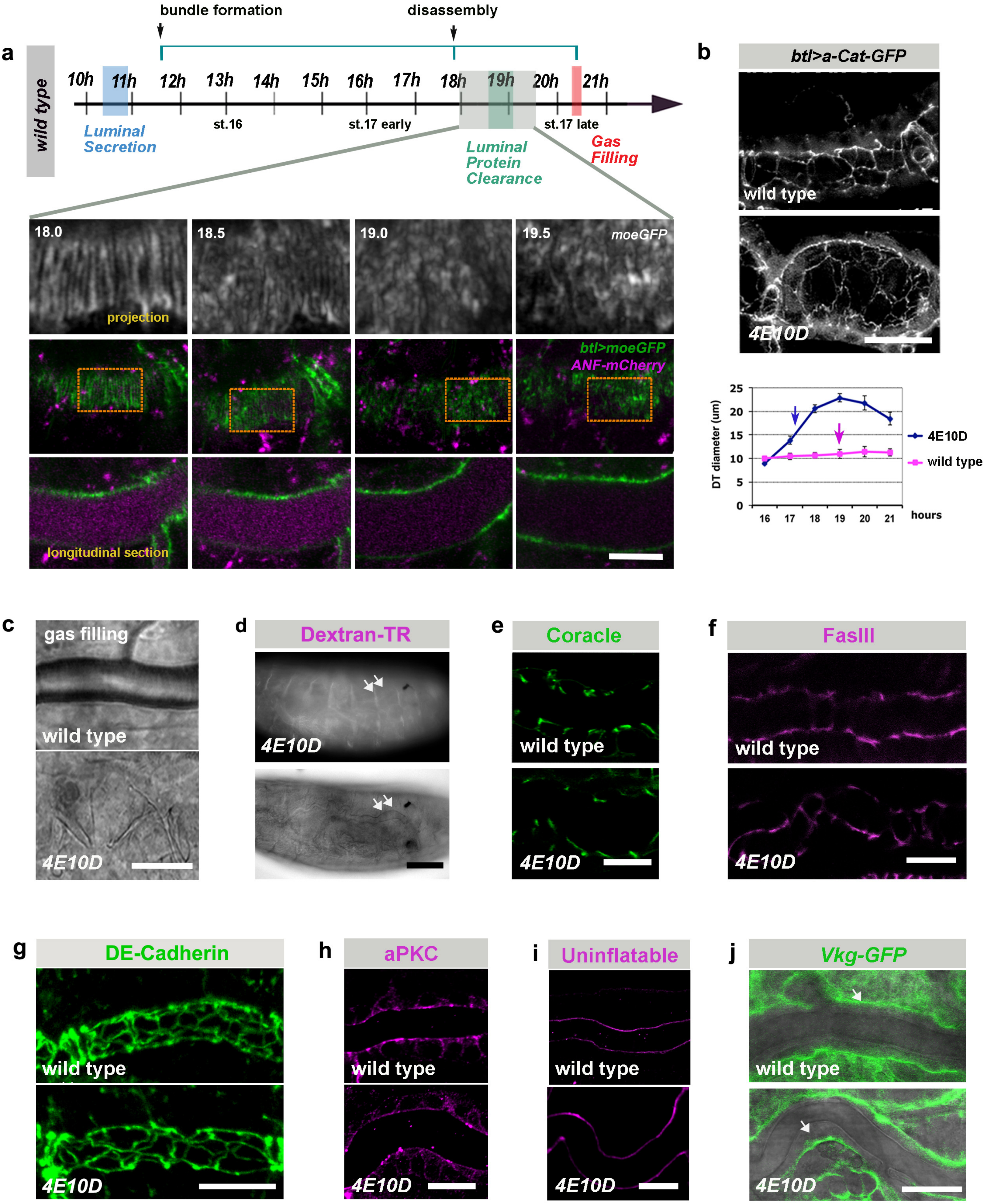

a
wild type
bundle formation
disassembly
10h
11h
12h
13h
14h
15h
16h
17h
18h
19h
20h
21h
st.16
st.17 early
st.17 late
Luminal Secretion
Luminal Protein Clearance
Gas Filling
18.0
18.5
19.0
19.5
moeGFP
projection
btl>moeGFP
ANF-mCherry
longitudinal section
b
btl>a-Cat-GFP
wild type
4E10D
DT diameter (um)
hours
4E10D
wild type
c
gas filling
wild type
4E10D
d
Dextran-TR
4E10D
e
Coracle
wild type
4E10D
f
FasIII
wild type
4E10D
g
DE-Cadherin
wild type
4E10D
h
aPKC
wild type
4E10D
i
Uninflatable
wild type
4E10D
j
Vkg-GFP
wild type
4E10D

Figure S1

Supplementary Figure 1. ***Ptp4EPtp10D*** **mutants show impaired tube growth but intact tracheal patterning and epithelial integrity.**

(a) Schematic representation of the perpendicular actin bundles in relation to the time of embryo development AEL (hours). Actin-bundles perpendicular to the tube axis (11.5-18.0 h AEL) are observed. A progressive loss of actin-bundles (18.0 h AEL and onwards) occurs concurrently to the initiation of luminal protein clearance in wild type embryos (green line, arrows).

Airyscan confocal of the DT in living wild-type embryos expressing *btl>moeGFP* (green) and *btl>ANFCherry* (magenta) (projections). Images in the upper row denote zoomed areas indicated by the rectangular frames. The conspicuous actin bundles (18.0h AEL) become weaker and turn into actin patches just before (18.5 h AEL) and during luminal clearance (19.0-19.5 h AEL) (longitudinal sections).

(b) DT in living late stage 17 wild-type and *Ptp4EPtp10D* embryos expressing *btl>α-Catenin-GFP* (grey scale). Diameter expansion rates of DT (Tr10) (16-21 h AEL) in wild-type (n = 5) and *Ptp4EPtp10D* (n = 8) mutant embryos expressing *btl-moeRFP* (graph panel). Time points of luminal protein clearance (arrows).

(c) Widefield images of a wild-type and *Ptp4EPtp10D* mutant embryo 21 hours (AEL). *Ptp4EPtp10D* mutants show gas filling defects and collapsed trachea.

(d) Fluorescent (upper) and widefield (lower) images of *Ptp4EPtp10D* mutant embryos injected with 10 kDa Dextran-TR at late stage 16 (15 h AEL). The dye was excluded from the tracheal lumen in wild-type (n = 37) or *Ptp4EPtp10D* (n = 36) (arrows) but was detected in *Mtf* mutants (n = 18) (negative control).

(e, f) Confocal images of the tracheal DT of wild-type and *Ptp4EPtp10D* mutant embryos stained for the septate junction components Coracle (green) (e) or Fasciclin III (FasIII, magenta) (f).

(g) Confocal images of the DT of living wild-type and *Ptp4EPtp10D* mutant embryos expressing endogenous DE-Cadherin-GFP (knock-in).

(h, i) Confocal images showing the DT of wild-type and *Ptp4EPtp10D* mutant embryos stained for the apical proteins, aPKC (magenta) (h) and Uninflatable (magenta) (i).

(j) Merged fluorescent/widefield images of living wild-type and *Ptp4EPtp10D* mutant embryos expressing the basal membrane marker for collagen type IV, Viking (Vkg)-GFP (green) (arrow). Scale bars, 10 µm (a-c, e-j), 50 µm (d). Embryos in (e) to (j) are late stage 16 to 17.

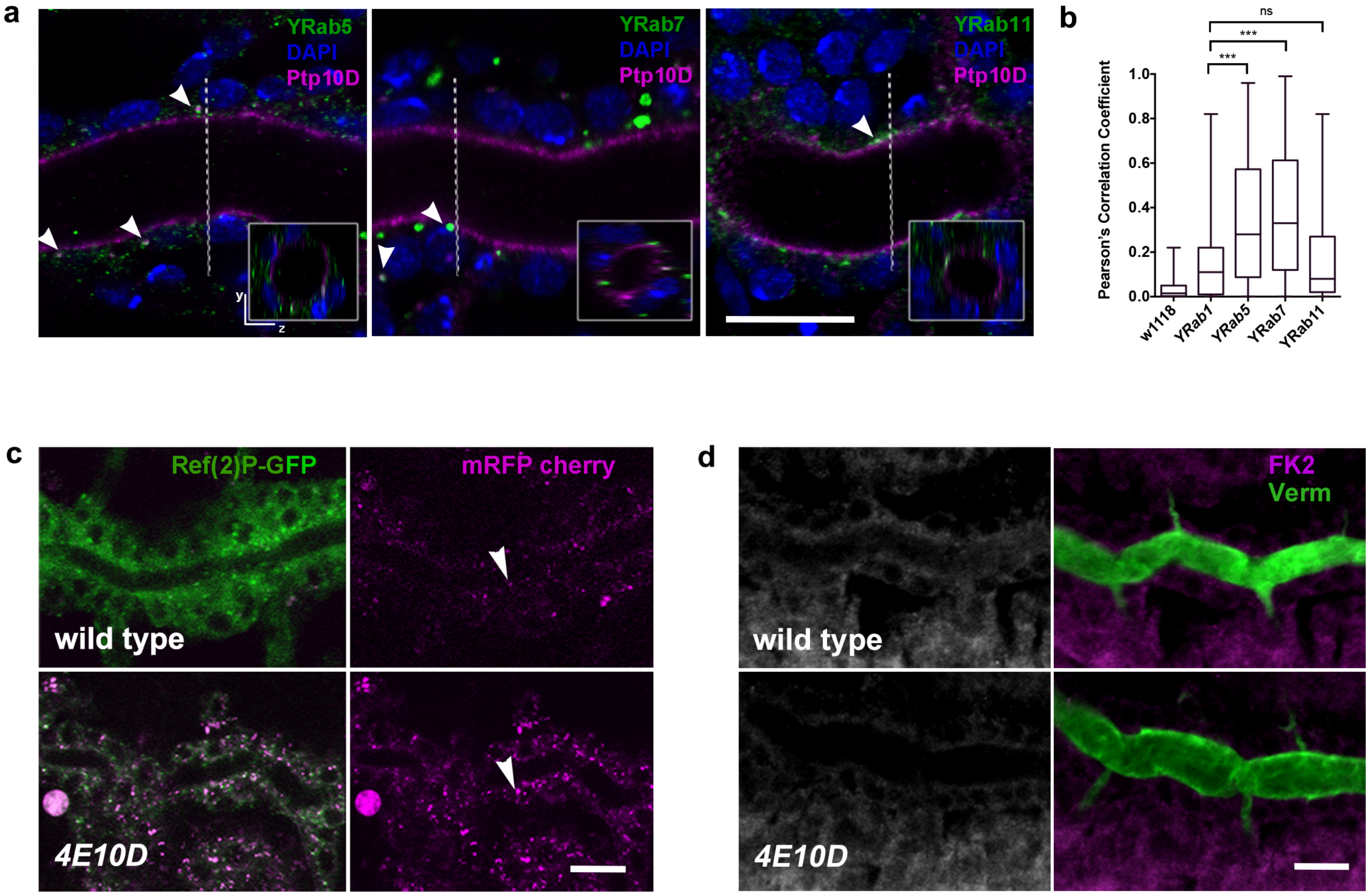

Supplementary Figure 2. ***Ptp4EPtp10D*** **mutants show enhanced lysosomal degradation.** (a) Confocal images of the tracheal DT in wild-type embryos expressing endogenous Rab-YFP (knock-in) proteins YFP-Rab5 or YFP-Rab7 or YFP-Rab11, stained with anti-Ptp10D (8B22F5, Cy3-magenta), anti-GFP (green) antibodies and DAPI (blue). Insets show cross section views of the DT (*y-z* plane) in the indicated DT position (vertical lines). Ptp10D localizes on the apical membrane and in the endocytic compartments of tracheal cells (arrowheads). (b) Pearson's correlation coefficient *r* of intracellular Cy3-Ptp10D puncta versus GFP signals in airways of w1118 (control of GFP antibody, *n* = 56 Ptp10D puncta, *n* =3 embryos) or YRab1 (*n* = 287 Ptp10D puncta, *n* = 9 embryos) or YRab5 (*n* =266 Ptp10D puncta, *n* = 10 embryos) or YRab7 (*n* = 346 Ptp10D puncta, *n* = 8 embryos) or YRab11 (*n* = 168 Ptp10D puncta, *n* = 6 embryos) embryos. Rab1 mediates membrane trafficking between ER and Golgi and used as a negative control of the endocytotic Rabs. ***, $P < 0.001$ (unpaired two tailed *t*-tests). *ns*, not significant ($P > 0.05$).
(c) Confocal sections of the tracheal DT of living stage 17 wild-type and *Ptp4EPtp10D* mutant embryos expressing the *Drosophila* p62 Ref(2)P-GFP-mCherry reporter. *Ptp4EPtp10D* mutants show high levels of mCherry (acidic compartments) but decreased GFP signal compared to wild-type. (d) Confocal images of the tracheal DT of early stage 17 wild-type and *Ptp4EPtp10D* mutant embryos stained for the ubiquitin marker FK2 (magenta) and the luminal protein Verm (green). Note the reduced ubiquitin signal in tracheal cells of *Ptp4EPtp10D* mutants in comparison to wild type. Scale bars, 10 µm.

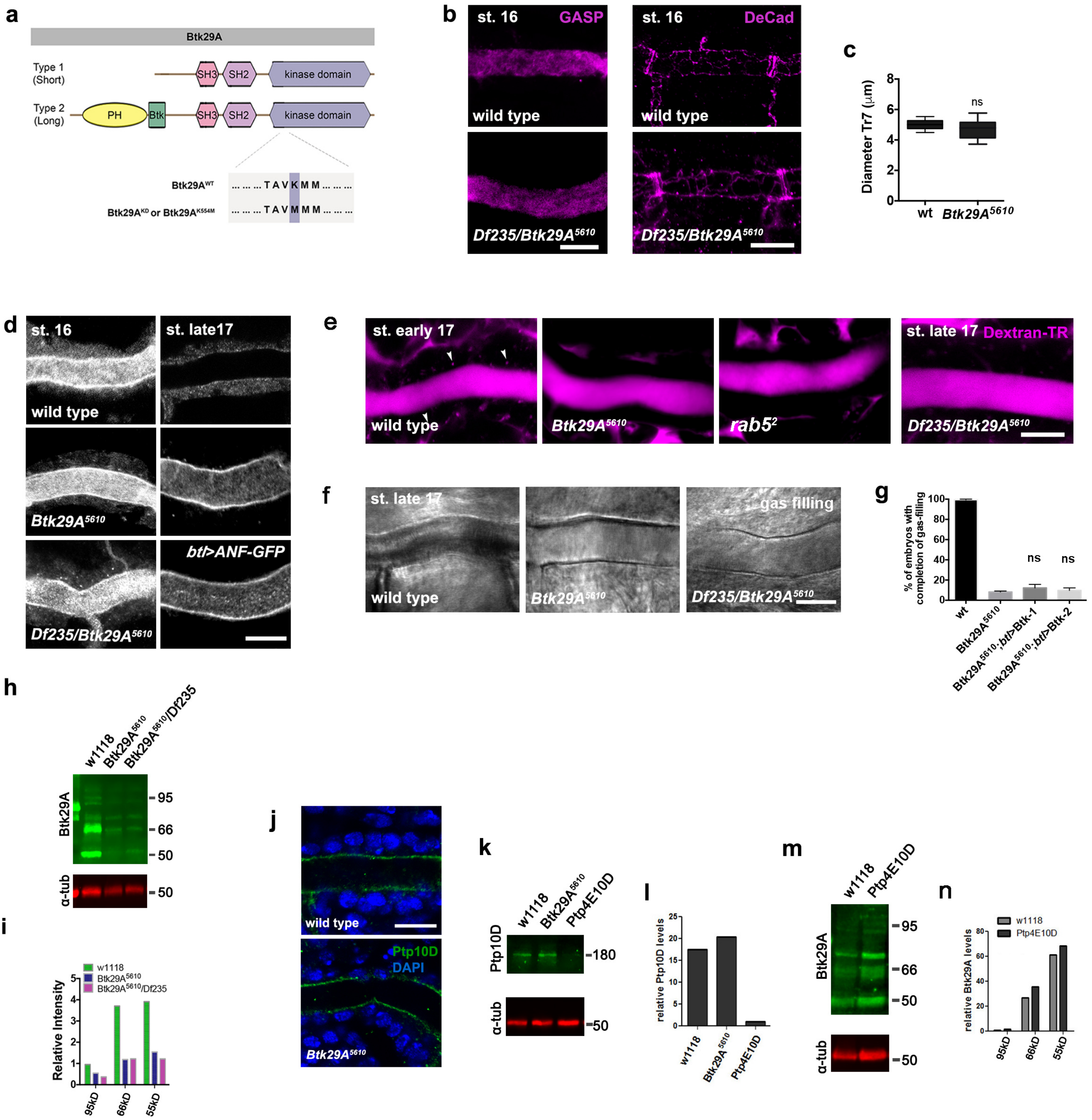

a
Btk29A
Type 1 (Short)
Type 2 (Long)
PH
Btk
SH3
SH2
kinase domain
Btk29A^WT ... ... T A V K M M ... ...
Btk29A^KD or Btk29A^K554M ... ... T A V M M M ... ...
b
st. 16
GASP
DeCad
wild type
Df235/Btk29A5610
c
Diameter Tr7 (μm)
ns
wt
Btk29A5610
d
st. 16
st. late17
wild type
Btk29A5610
Df235/Btk29A5610
btl>ANF-GFP
e
st. early 17
st. late 17
Dextran-TR
wild type
Btk29A5610
rab5²
Df235/Btk29A5610
f
st. late 17
gas filling
wild type
Btk29A5610
Df235/Btk29A5610
g
% of embryos with completion of gas-filling
ns
wt
Btk29A5610
Btk29A5610;btl>Btk-1
Btk29A5610;btl>Btk-2
h
w1118
Btk29A5610
Btk29A5610/Df235
Btk29A
α-tub
95
66
50
i
Relative Intensity
w1118
Btk29A5610
Btk29A5610/Df235
95kD
66kD
55kD
j
wild type
Btk29A5610
Ptp10D
DAPI
k
w1118
Btk29A5610
Ptp4E10D
Ptp10D
α-tub
180
50
l
relative Ptp10D levels
w1118
Btk29A5610
Ptp4E10D
m
w1118
Ptp4E10D
Btk29A
α-tub
95
66
50
n
relative Btk29A levels
w1118
Ptp4E10D
95kD
66kD
55kD

Figure S3

Supplementary Figure 3. Btk29A is required for luminal endocytosis.

(a) Schematic representation of *Drosophila* Btk29A protein. Btk29A consists of SH3, SH2 and kinase domain (Type 1 or Short isoform), a BTK domain and a PH domain (Type 2 or Long isoform). Domains shown: pleckstrin homology (PH); Btk homology region (Btk); Src-homology 3 (SH3); Src-homology 2 (SH2); tyrosine kinase domain. The single amino acid substitution in the kinase domain (K554M) of the Type 2 (or long isoform) is indicated.

(b) Confocal images of the tracheal DT of wild-type and $Df235/Btk29A^{5610}$ mutant embryos stained for the luminal antigen GASP (magenta, upper row) or DE-Cadherin (magenta, lower row). (c) Plots showing the average diameter (Tr7) of stage 16 wild-type (n = 13) and $Btk29A^{5610}$ embryos (n = 14). *ns*, not significant ($P > 0.05$), (unpaired two tailed *t*-tests).

(d) Confocal images showing the DT of stage 16 and late stage 17 wild-type, $Btk29A^{5610}$ and $Df235/Btk29A^{5610}$ mutant embryos expressing *btl>ANF-GFP*. Embryos were stained with anti-GFP (gray scale). (e) Confocal frames showing the DT of living wild-type, $Btk29A^{5610}$, $rab5^2$ and D$f235/Btk29A^{5610}$ embryos subjected to Dextran-TR endocytic assay. Images depict tracheal DT, just before (first three images) and after (last image) luminal clearance. (f) Wide-field images showing the gas filling defects in $Btk29A^{5610}$ and $Df235/Btk29A^{5610}$ compared to wild type.

(g) Plots showing the percentage of wild-type (n = 54), $Btk29A^{5610}$ (n = 67), $Btk29A^{5610}$*;btl>Btk29A-1* (type-1) (n = 58) and $Btk29A^{5610}$ *;btl>Btk29A-2* (type-2) (n = 72) embryos that complete gas-filling. *ns*, $P > 0.05$ (unpaired two tailed *t*-tests). (h, i) Western blot analysis of wild-type, $Btk29A^{5610}$ and $Btk29A^{5610}/Df235$ mutant embryos using a rat anti-Btk29A antibody (h). The Btk29A/α-tubulin signal ratio was calculated for each of the 3 isoforms (95, 66 and 55 kDa) (i). The western blot signal quantification was measured with the presentative blot (h) from at least 3 independent experiments. (j) Confocal images of the tracheal DT of $Btk29A^{5610}$ embryos stained for Ptp10D (green) and DAPI (blue). (k, l) Western blot analysis of Ptp10D (8B22F5 and 45E10, antibody mix) in wild-type, $Btk29A^{5610}$ and *Ptp4EPtp10D* mutants. α-tubulin (loading control) (k). Ptp10D/α-tubulin signal ratio (l). The western blot signal quantification refers to presentative blot (k). This outcome was reproducible in three independent Western blot experiments (n = 3). (m, n) Western blot analysis of Btk29A in wild-type and *Ptp4EPtp10D* mutant embryos. α-tubulin (loading control) (m). The Btk29A/α-tubulin signal ratio (n). The western blot signal quantification refers to the presentative blot (m) and it was reproducible in 3 independent experiments (n = 3). Scale bars, 10 μm.

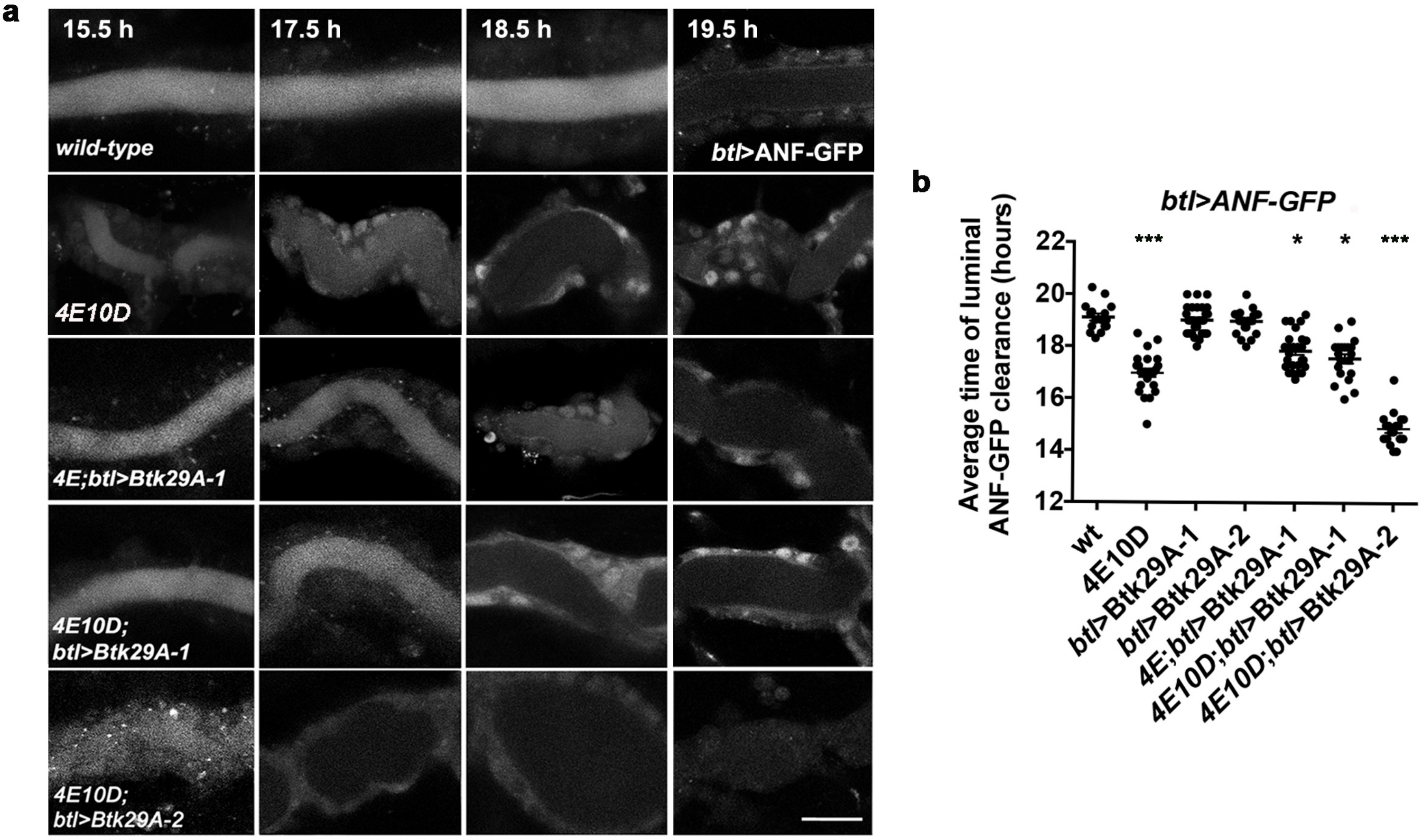

Supplementary Figure 4. PTPs antagonize Btk29A during luminal clearance.

(a) Confocal frames showing the DT of living wild-type, *Ptp4EPtp10D, Ptp4E,btl>Btk29A-1* (type-1)*, Ptp4EPtp10D,btl>Btk29A-1* (type-1) and *Ptp4EPtp10D,btl>Btk29A-2* (type-2) embryos expressing *btl*>ANF-GFP (grey scale). *Ptp4E* mutants (but not wild-type) embryos expressing Btk29A in the tracheal cells show over-elongated DT and premature luminal ANF-GFP clearance. These phenotypes are enhanced in *Ptp4EPtp10D;btl>Btk29A-2* embryos. Time points depict hours AEL.

(b) Plots depicting the average time (hours) of ANF-GFP luminal clearance in wild-type (n = 17), *Ptp4EPtp10D (n =24), btl>Btk29A-1* (n = 28), *btl>Btk29A-2* (n = 25), *Ptp4E,btl>Btk29A-1* (n = 26), *Ptp4EPtp10D,btl>Btk29A-1* (n = 17) and *Ptp4EPtp10D,btl>Btk29A-2* (n = 18) embryos. *Ptp4E,btl>Btk29A-1* activated luminal ANF-GFP clearance 56 min earlier. Scatter plot shows data points and corresponding mean (horizontal line). Unpaired two tailed t-tests were performed in relation to wild-type data set of experimental mean values. *, $P < 0.012$, ***, $P < 0.0001$, (unpaired two tailed t-tests).

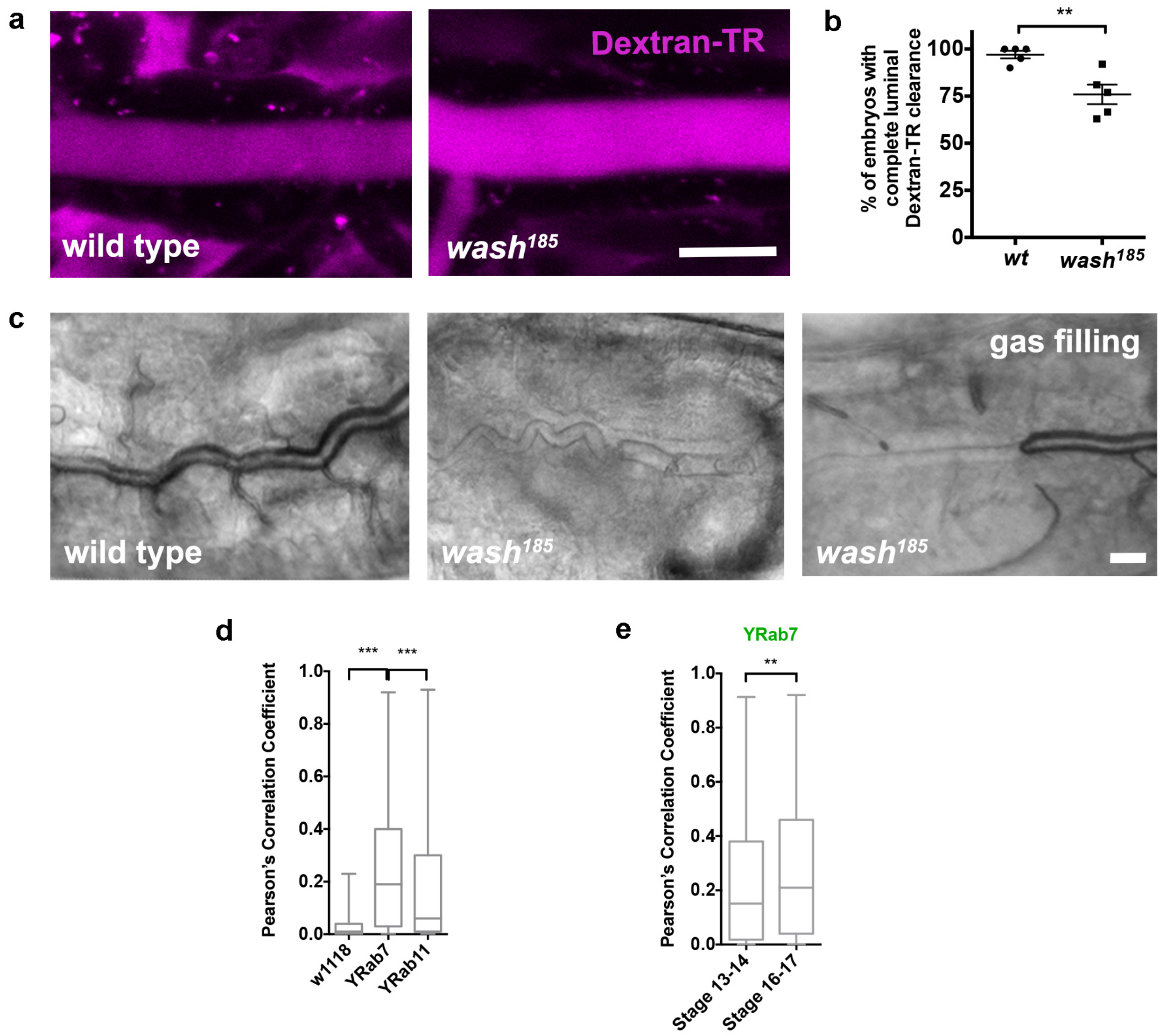

Supplementary Figure 5. WASH is required for luminal endocytosis and gas filling in *Drosophila* airways.

(a) Confocal frames of the tracheal DT of wild-type and *wash*^{185} embryos (18.5 hours AEL) subjected to Dextran-TR endocytic assay. Note that intracellular Dextran-TR puncta are reduced in *wash*^{185} mutant embryos.

(b) Scatter plots depicting the percentage of embryos (%) with luminal Dextran-TR clearance completion in wild type (n = 12) and *wash*^{185} (n = 18) embryos. **, $P < 0.005$. (c) Wide-field images of living stage 17 wild-type and *wash*^{185} embryos (21.5 h AEL). *wash*^{185} mutants showed partial or no gas-filling. (d) Pearson's correlation coefficient r of intracellular WASH puncta and GFP signals in airways of w1118 (n = 166 WASH puncta, n =3 embryos) (as control of GFP antibody) or YRab7 (n = 359 WASH puncta, n = 5 embryos) or YRab11 (n = 356 WASH puncta, n = 5 embryos). ***, $P < 0.001$ (unpaired two tailed t-tests). *ns*, not significant ($P > 0.05$). (e) Pearson's correlation coefficient r of intracellular WASH puncta and GFP signals in airways of embryos expressing YRab7 during stage 13-14 (n = 244 WASH puncta, n = 5 embryos) and late16 -17 stages (n = 355 WASH puncta, n = 5 embryos). **, $P < 0.01$ (unpaired two tailed t-tests). The boxplots (d, e) show the median (horizontal line) and the data range from 25th to 75th percentile. Bars present maxima and minima values. Scale bars, 10 µm.

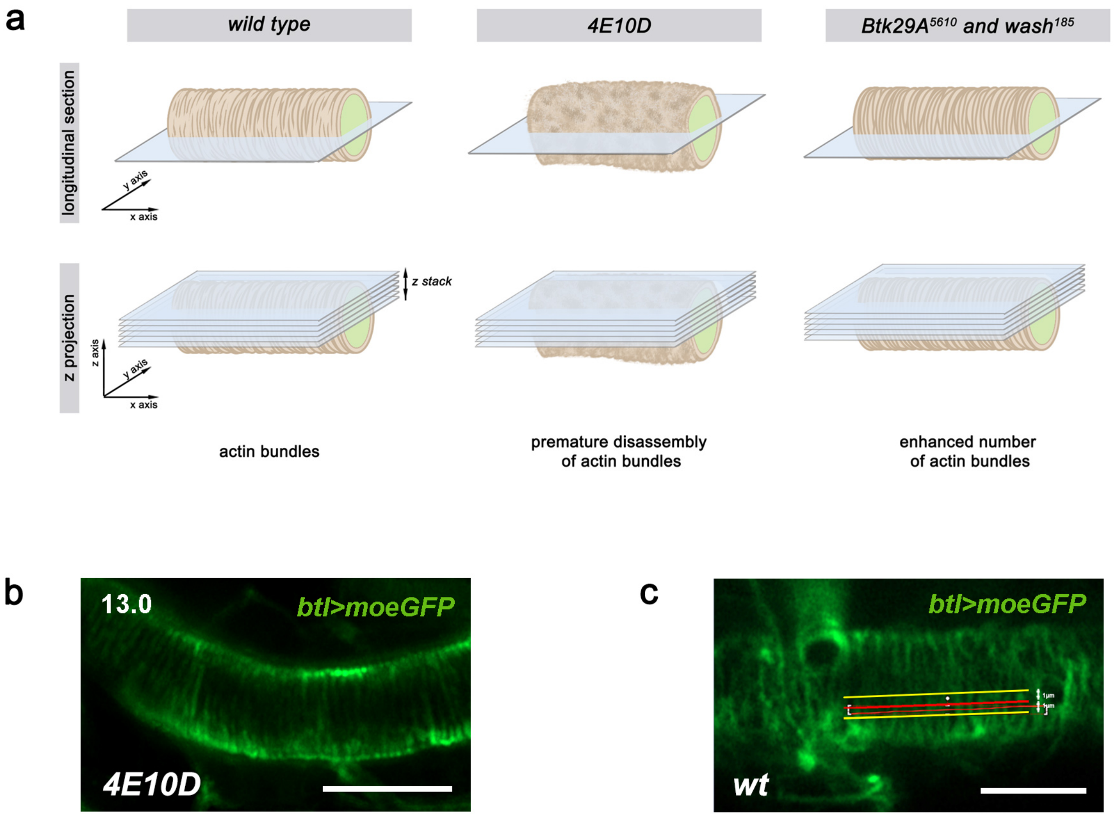

Supplementary Figure 6. Apical actin bundles in *Ptp4EPtp10D, Btk29A*^{5610} and *wash*^{185}.

(a) Schematic overview of the actin bundles phenotypes depicted in Fig. 5. Wild type, *Btk29A*^{5610} and *wash*^{185} embryos were examined 18 hours AEL (just before luminal clearance) and *Ptp4EPtp10D* 17 hours AEL (just before the premature luminal clearance), using airyscan super-resolution confocal microscopy. Single sections (upper row) were used for the visualization of the apical actin and Z-stack maximum projections (lower row) for the visualization of the actin bundles. Note that the actin bundles observed in wild-type, *Btk29A*^{5610} and *wash*^{185} embryos are dissociated in *Ptp4EPtp10D* much earlier, concurrently to the premature luminal clearance.

(b) Z-projection showing the DT of a *Ptp4EPtp10D* embryo expressing *btl>moe-GFP* (green) 13 hours AEL. The apical actin bundles are intact at this stage in *Ptp4EPtp10D* embryos.

(c) Z-projection of a wild-type embryo expressing *btl>moe-GFP* (green) 18 h AEL. Three horizontal lines along the tube axis, 1µm apart, were drawn. Actin bundles $\geq$ 2 µm long were quantified. Scale bar 10µm.

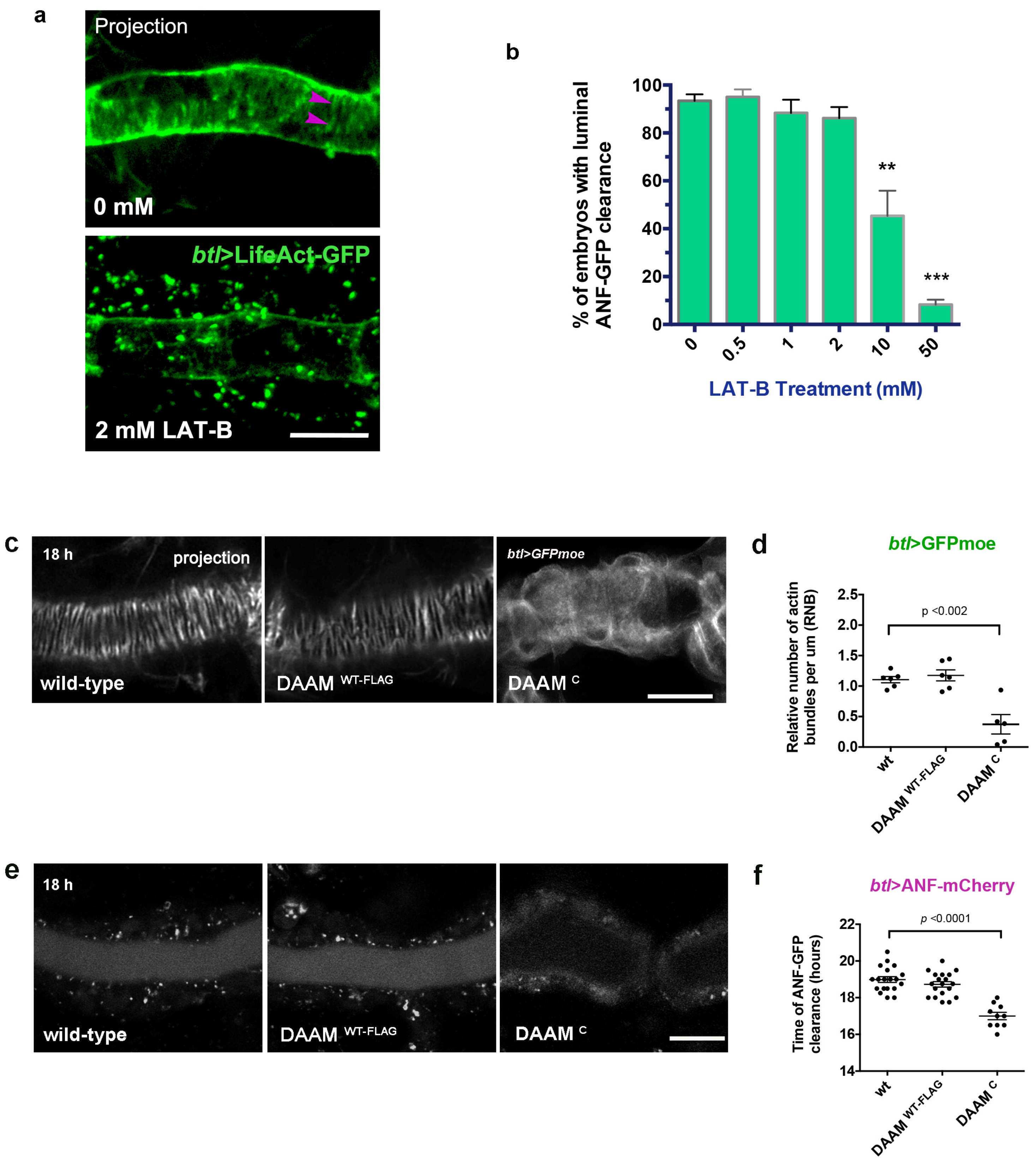
a
Projection
0 mM
btl>LifeAct-GFP
2 mM LAT-B
b
% of embryos with luminal ANF-GFP clearance
100
80
60
40
20
0
**
***
0
0.5
1
2
10
50
LAT-B Treatment (mM)
c
18 h
projection
wild-type
DAAM WT-FLAG
btl>GFPmoe
DAAM C
d
btl>GFPmoe
Relative number of actin bundles per um (RNB)
p <0.002
2.5
2.0
1.5
1.0
0.5
0.0
wt
DAAM WT-FLAG
DAAM C
e
18 h
wild-type
DAAM WT-FLAG
DAAM C
f
btl>ANF-mCherry
Time of ANF-GFP clearance (hours)
p <0.0001
22
20
18
16
14
wt
DAAM WT-FLAG
DAAM C

Figure S7

Supplementary Figure 7. Chemical disruption of the actin bundles affects tube clearance in a dose dependent manner.

(a) Confocal projections of the DT in wild-type embryos expressing *btl>LifeAct-GFP* showing actin bundles before (upper image) and after treatment with 2 mM Latrunculin–B (LAT-B) (lower image). LAT-B treatment induces actin bundle losses, coupled with an increase of actin patches. Representative images from 3 independent experiments are shown.

(b) Plots showing the percentage of wild-type embryos treated with different concentrations of LAT-B (total $n = 148$) that complete luminal ANF-GFP clearance. Note that high concentrations of LAT-B block luminal ANF-GFP clearance. **, $P < 0.002$; ***, $P < 0.0001$ (unpaired two tailed *t*-tests).

c) Confocal projections of the DT of wild-type, *btl>DAAM* $^{WT\text{-}FLAG}$*,* and *btl>DAAM* ^{C} embryos expressing the actin reporter *btl>moeGFP* (grey). Images were acquired, 18h AEL. Actin bundles in *btl>DAAM* ^{C} were disrupted.

(d) Plots showing the relative number of actin bundles (> 2 µm long) per *µm* (RNB) in wild-type (n =6), *DAAM* $^{WT\text{-}FLAG}$ *(*n = 6)*,* and *DAAM* ^{C} (n =5) embryos expressing *btl>moeGFP*. **, $P <$ 0.002 (unpaired two tailed *t*-tests).

(e) Confocal frames showing the DT of living wild-type, *btl>DAAM* $^{WT\text{-}FLAG}$*,* and *btl>DAAM* ^{C} embryos expressing *btl>ANF-mCherry* (grey). Luminal ANF-mCherry is prematurely cleared in *btl>DAAM* ^{C} embryos.

(f) Plots showing the average time (hours) of luminal ANF-GFP clearance of wild-type (n =19), *btl>DAAM* $^{WT\text{-}FLAG}$ (n =19)*,* and *btl>DAAM* ^{C} (n =10) embryos. **, $P < 0.0001$ (unpaired two tailed *t*-tests).

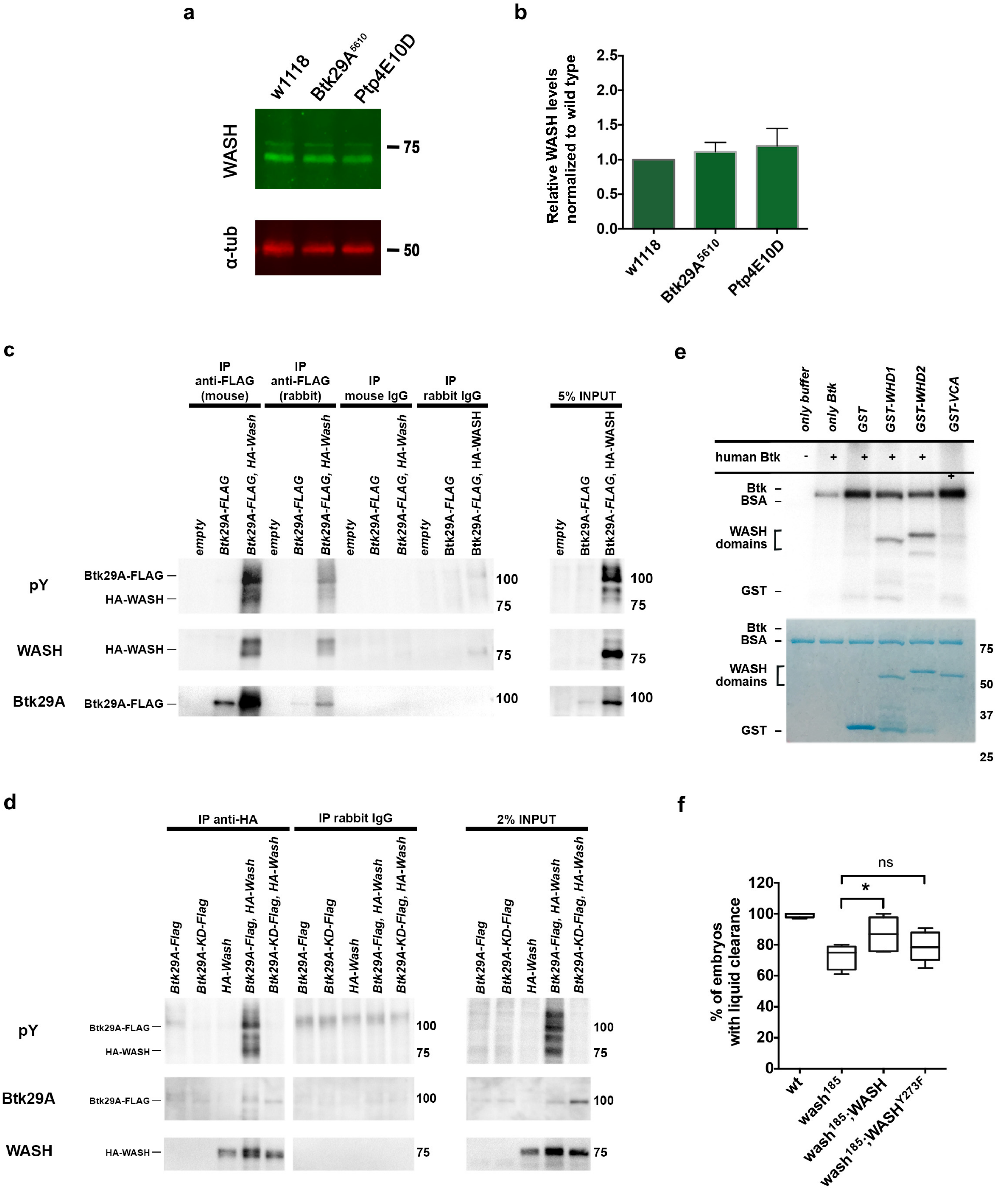
a
w1118
Btk29A5610
Ptp4E10D
WASH
75
α-tub
50
b
Relative WASH levels normalized to wild type
2.5
2.0
1.5
1.0
0.5
0.0
c
IP anti-FLAG (mouse)
IP anti-FLAG (rabbit)
IP mouse IgG
IP rabbit IgG
5% INPUT
empty
Btk29A-FLAG
Btk29A-FLAG, HA-Wash
pY
Btk29A-FLAG
HA-WASH
WASH
Btk29A
100
75
d
IP anti-HA
IP rabbit IgG
2% INPUT
Btk29A-Flag
Btk29A-KD-Flag
HA-Wash
Btk29A-Flag, HA-Wash
Btk29A-KD-Flag, HA-Wash
e
only buffer
only Btk
GST
GST-WHD1
GST-WHD2
GST-VCA
human Btk
Btk
BSA
WASH domains
GST
75
50
37
25
f
% of embryos with liquid clearance
120
100
80
60
40
20
0
ns
*
wt
wash185
wash185;WASH
wash185;WASHY273F

Figure S8

Supplementary Figure 8. WASH can be phosphorylated and is a conserved substrate of Btk29A.

(a) Western blot analysis showing the expression levels of WASH in lysates of wild type, *Ptp4EPtp10D and* $Btk29A^{5610}$ embryos.

(b) Plots depicting the relative WASH/α-tubulin signals shown in (a), normalized to wild type. The western blot signal quantification derived from 3 independent experiments.

(c) Co-immunoprecipitates of FLAG tagged Btk29A protein from lysates of transfected S2 cells (using two different anti-FLAG antibodies and mouse or rabbit IgG as control), blotted with anti-pY, anti-WASH and anti-Btk29A. The cells were transfected with pAWF-*Btk29A* and pAHW-*wash* vectors as indicated. Btk29A-FLAG interacts with HA-WASH and phosphorylation of both proteins was observed (75 and 100kDa for HA-WASH and Btk29A-FLAG respectively). The same result was acquired in two independent repetitions of the experiment.

(d) Co-immunoprecipitates of WASH from lysates of transfected S2 cells (using anti-HA antibody or rabbit IgG as a control), blotted with anti-pY, anti-Btk29A and anti-WASH. S2 cells were transfected with pAWF vector containing *Btk29A* or $Btk29A^{KD}$ and pAHW containing *Wash* cDNA or combinations of the above (as indicated). HA-WASH interacts with both Btk29A-FLAG and Btk29AKD-FLAG. A strong 75kDa pY signal is detected only when HA-WASH and Btk29A-FLAG are co-expressed suggesting that Btk29A is sufficient to phosphorylate WASH protein. The same results were acquired in three independent repetitions of the experiment.

(e) *In vitro* phosphorylation assay of the human WASH domains (WHD1, WHD2 and VCA) using 100ng of human Btk kinase. The γ-^{32}P signal was detected with autoradiography (upper panel) and the respective protein amounts were estimated by Coomassie blue staining (lower panel).

(f) Plots depicting the percentage of wild-type (n = 21), $wash^{185}$ (n = 27), $wash^{185}$*;btl> Wash* (n = 31) and $wash^{185}$ *;btl>* $Wash^{Y273F}$ (n = 39) embryos that complete gas-filling. $wash^{185}$ embryos expressing the *Wash* but not the non-phosphorylatable variant $Wash^{Y273F}$, restored the gas-filling defects of $wash^{185}$ mutants. *, $P < 0.03$ (unpaired two tailed t-tests).

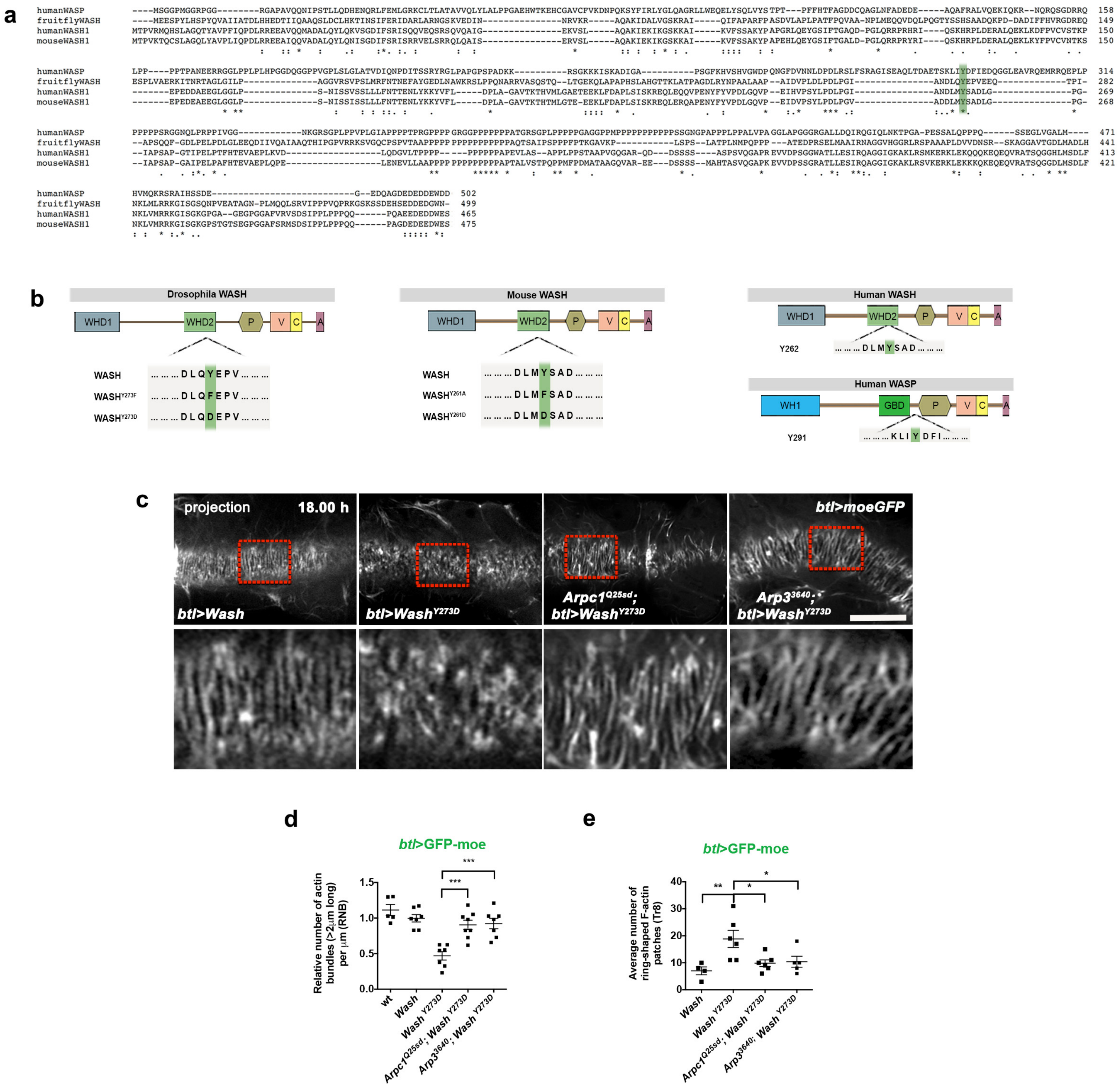

Figure S9

Supplementary Figure 9. The Y^{273} residue of WASH.

(a) Comparative protein sequence alignment of human Wasp, human WASH, mouse WASH and *Drosophila* WASH. The Wasp tyrosine residue 291 (Y^{291}), known as a substrate of Src kinases, is conserved in *Drosophila*, human and mouse WASH protein (Y^{273}, Y^{262} and Y^{261} respectively) (green box).

(b) Schematic representation of *Drosophila* WASH, mouse WASH, human WASH and human Wasp proteins. The single amino acid substitutions of WASH tyrosine residue Y273 to Y273F or Y273D and the corresponding Y261 of mouse WASH, Y262 of human WASH, and Y291 of human Wasp are indicated (in green). WH1: Wasp homology region 1; GBD/CRIB: GTPase binding/Cdc42 and Rac interactive binding domain; WHD1: WASH homology domain 1; WHD2: WASH homology domain 2; P: proline-rich domain; V: Verprolin homologous domain; C: connecting or central domain; A: acidic domain. V, C and A together are referred to as the VCA domain.

(c) Airyscan confocal images of the DT of wild-type, *btl>Wash* or *btl>*$Wash^{Y273D}$, $Arpc1^{Q25sd}$*; btl>*$Wash^{Y273D}$ and $Arp3^{3640}$*;btl>*$Wash^{Y273D}$ embryos expressing the actin cytoskeleton reporter *moe-GFP* (grey). Images were acquired during luminal endocytosis, 18 h AEL. Z-stack projections are shown. Lower row depicts zoomed view of areas indicated by the rectangular frames (red). Scale bars, 10µm.

(d) Scatter plots showing the relative number of actin bundles (> 2 µm long) per *µm* (RNB) in wild-type (n = 5), *btl>Wash* (n = 7) or *btl>*$Wash^{Y273D}$ (n = 7), $Arpc1^{Q25sd}$*; btl>*$Wash^{Y273D}$ (n = 8) and $Arp3^{3640}$*;btl>*$Wash^{Y273D}$ (n = 7) embryos expressing *btl>moeGFP*. *** indicate statistical significance $P < 0.0005$ respectively, in comparison to wild-type (unpaired two tailed *t*-tests).

(e) Scatter plots showing the average number of ring-shaped actin patches in *btl>Wash* (n = 4) or *btl>*$Wash^{Y273D}$ (n = 6) or $Arpc1^{Q25sd}$*; btl>*$Wash^{Y273D}$ (n = 6) or $Arp3^{3640}$*;btl>*$Wash^{Y273D}$ (n = 5) embryos. **, $P < 0.01$, *$P < 0.03$ (unpaired two tailed *t*-tests).

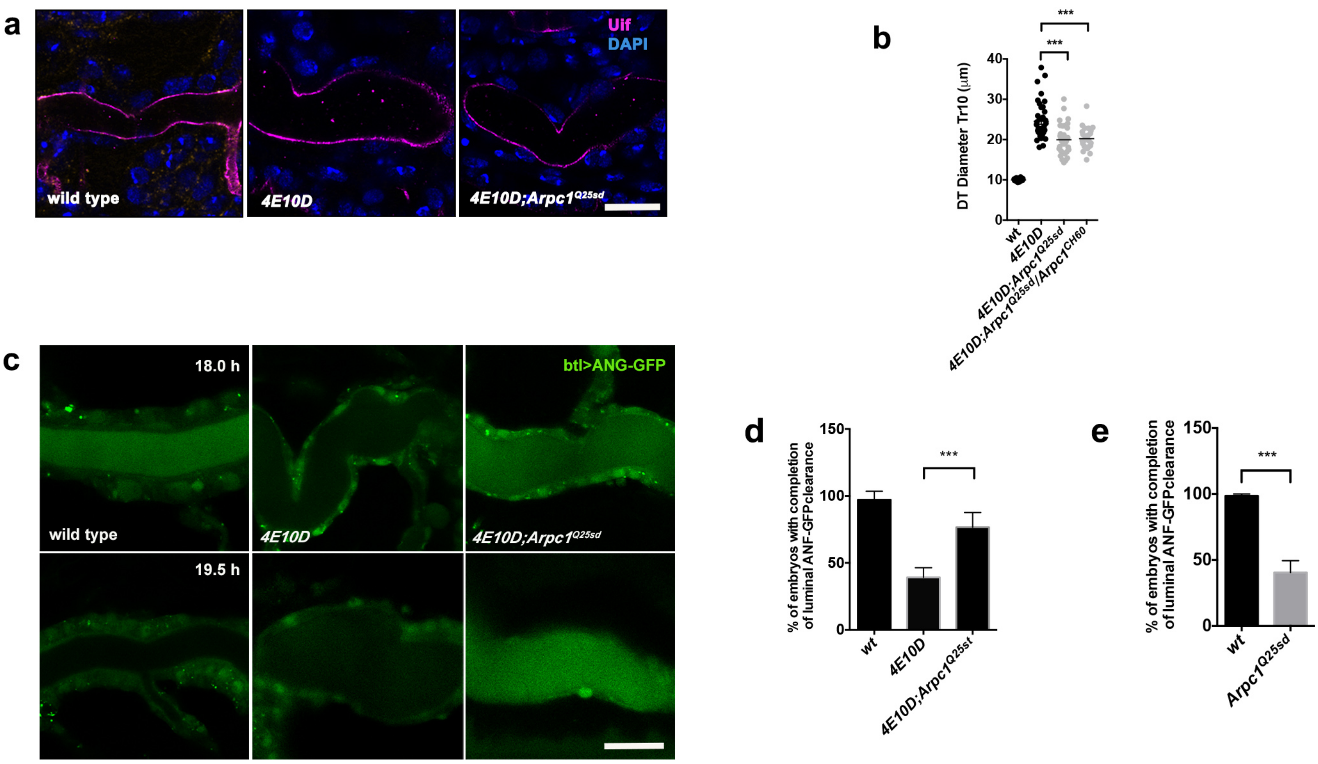

Supplementary Figure 10. Arpc1 mutations suppress *Ptp4EPtp10D* phenotypes.
(a) Confocal images of the tracheal DT of wild-type, *Ptp4EPtp10D* and *Ptp4EPtp10D; $Arpc1^{Q25s}$* mutant embryos (stage 17) stained for the apical marker Uif (magenta) and DAPI (blue). The reduction of *Arpc1* in *Ptp4EPtp10D* mutants suppressed the tube shape defects. (b) Plots depicting the average diameter of the DT (Tr8) in wild-type (n = 10), *Ptp4EPtp10D* (n = 39), *Ptp4EPtp10D; $Arpc1^{Q25sd}$* (n = 35) and *Ptp4EPtp10D; $Arpc1^{Q25sd}$/ $Arpc1^{CH60}$* (n = 24) embryos. The DT diameter phenotype of *Ptp4EPtp10D* is significantly suppressed in *Ptp4EPtp10D; $Arpc1^{Q25sd}$* or in *Ptp4EPtp10D; $Arpc1^{Q25sd}$/ $Arpc1^{CH60}$* mutants. Unpaired two-tailed t-test. ***, P <0.001. (c) Confocal frames showing the DT of living wild-type, *Ptp4EPtp10D* and *Ptp4EPtp10D; $Arpc1^{Q25sd}$* mutant embryos expressing *btl>ANF-GFP* (green), 18 h AEL (before luminal clearance) and 19.5 h AEL (after the luminal clearance). *Ptp4EPtp10D* clear earlier ANF-GFP (18h), while *Ptp4EPtp10D; $Arpc1^{Q25sd}$* mutant embryos retain luminal ANF-GFP at 19.5 h (lower row). Time points represent hours AEL. (d) Bar graph showing the percentage of wild-type (n = 27), *Ptp4EPtp10D* (n = 38) and *Ptp4EPtp10D; $Arpc1^{Q25sd}$* (n = 36) embryos expressing *btl>ANF-GFP*, that completed luminal protein clearance at 18 h AEL. ***, P <0.0005. Plots show mean values and s.e.m of five independent experiments. Unpaired two-tailed t-test. (e) Bar graph showing the percentage of wild-type (n = 26) and *$Arpc1^{Q25sd}$* (n = 44) embryos that completed luminal ANF-GFP protein clearance. Plots show mean values and s.e.m of five experiments. ***, P <0.0005. Unpaired two-tailed t-test.

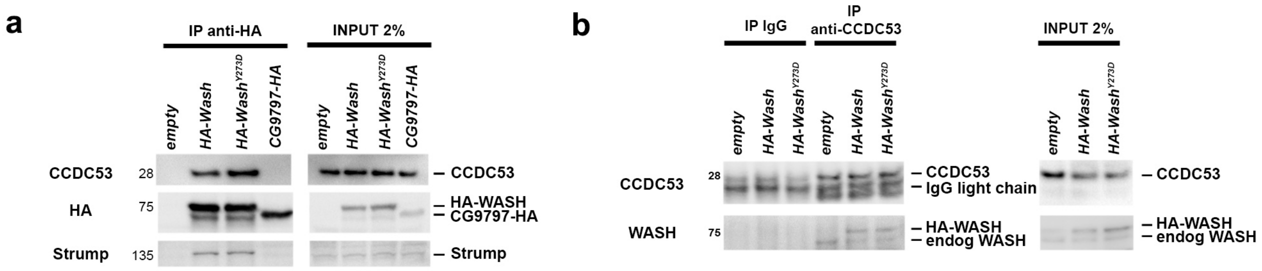

Supplementary Figure 11. Wash^{Y273D} mutation does not disrupt the interactions of WASH with its regulatory complex.

(a) Co-immunoprecipitates of HA-tagged WASH protein from lysates of transfected S2 cells (using anti-HA affinity matrix), blotted with anti-CCDC53, anti-HA and anti-Strumpellin. The cells were transfected with pAHW containing *Wash* or *Wash^{Y273D}*, or pAWH containing *CG9797* (as a negative control) cDNA, as indicated.

(b) Co-immunoprecipitates of endogenously expressed CCDC53 protein from lysates of transfected S2 cells, blotted with anti-CCDC53 and anti-WASH. The cells were transfected with pAHW containing *Wash* or *Wash^{Y273D}* cDNA, as indicated.

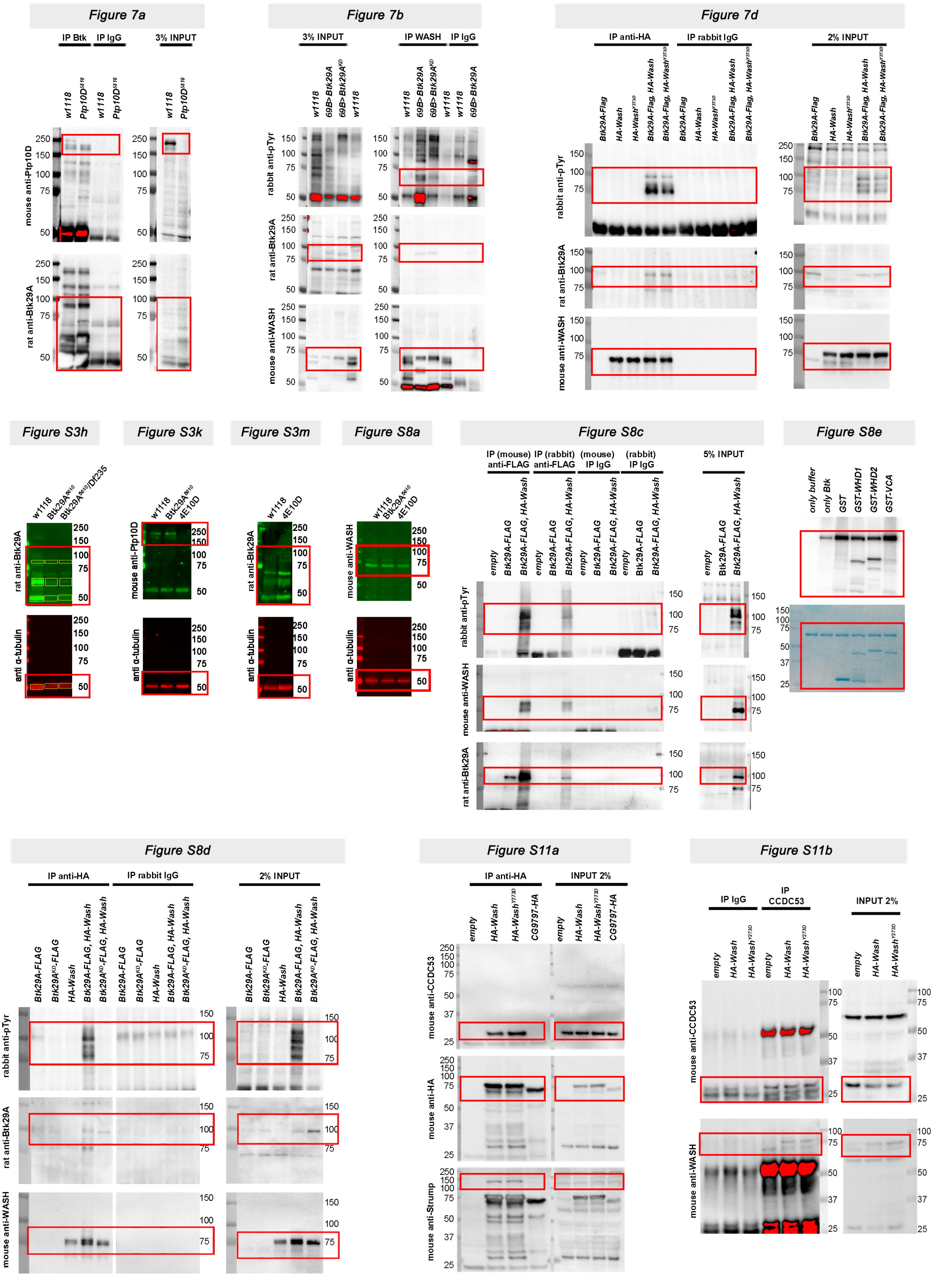
Figure 7a
IP Btk
IP IgG
3% INPUT
w1118
Ptp10D
mouse anti-Ptp10D
rat anti-Btk29A
Figure 7b
3% INPUT
IP WASH
IP IgG
w1118
69B>Btk29A
rabbit anti-pTyr
rat anti-Btk29A
mouse anti-WASH
Figure 7d
IP anti-HA
IP rabbit IgG
2% INPUT
Btk29A-Flag
HA-Wash
Btk29A-Flag, HA-Wash
rabbit anti-pTyr
rat anti-Btk29A
mouse anti-WASH
Figure S3h
Figure S3k
Figure S3m
Figure S8a
w1118
4E10D
rat anti-Btk29A
mouse anti-Ptp10D
mouse anti-WASH
anti α-tubulin
Figure S8c
IP (mouse) anti-FLAG
IP (rabbit) anti-FLAG
(mouse) IgG
(rabbit) IgG
5% INPUT
empty
Btk29A-FLAG
Btk29A-FLAG, HA-Wash
rabbit anti-pTyr
mouse anti-WASH
rat anti-Btk29A
Figure S8e
only buffer
only Btk
GST
GST-WHD1
GST-WHD2
GST-VCA
Figure S8d
IP anti-HA
IP rabbit IgG
2% INPUT
Btk29A-FLAG
HA-Wash
Btk29A-FLAG, HA-Wash
rabbit anti-pTyr
rat anti-Btk29A
mouse anti-WASH
Figure S11a
IP anti-HA
INPUT 2%
empty
HA-Wash
CG9797-HA
mouse anti-CCDC53
mouse anti-HA
mouse anti-Strump
Figure S11b
IP IgG
IP CCDC53
INPUT 2%
empty
HA-Wash
mouse anti-CCDC53
mouse anti-WASH

Figure S12

Supplementary Figure 12. Unprocessed Western blots.

Unprocessed original blots used in the main and supplementary figures. Red boxes indicate the region presented in the figures. The corresponding figure is indicated on the top of each plot. The yellow frames in figure S3h indicate the blot regions used to quantify intensities.

Supplementary Table 1. Genetic interaction between *Ptp4EPtp10D* and mutants of other RTK or TK components.

	Genotype	Tube diameter (μm) [a]	Statistical significance	*n*
		mean		
**Receptor**	w^{1118} (wild type)	9.49	-	*52*
**Tyrosine kinases**	*$4E^{1}10D^{1}$*	19.5	*** [b]	*72*
**(RTKs)**	*4E10D; btl>$EGFR^{DN}$*	19.6	ns	*52*
	4E10D; btl>$EGFR^{E1}$	20.8	ns	*24*
	4E10D; $EGFR^{k05115}$/+	20.5	ns	*53*
	4E10D; btl>$FGFR^{\lambda TOP}$	18.9	ns	*38*
	4E10D; $stitcher^{Excel9056}$	20.1	ns	*28*
	4E10D; btl>$stitcher^{KD}$	18.9	ns	*42*
	4E10D; btl>$stitcher^{CA}$	20.87	ns	*51*
	4E10D; pvr^{c02195}	20.27	ns	*22*
	4E10D; pvr^{c02859}	19.15	ns	*25*
**Tyrosine Kinases**	*4E10D; $Src42A^{E1}$*	18.8	ns	*57*
**(TKs)**	*4E10D; $Src42A^{DN}$*	17.1	ns	*59*
	4E10D; Src64B	19.5	ns	*48*
	4E10D; $Src42A^{CA}$	20.6	ns	*39*
	4E10D; $Btk29A^{5610}$	11.8	*** [c]	*27*
	4E10D; $Src42A^{E1}$ $Btk29A^{5610}$	12.5	*** [c]	*64*

[a] The diameter of DT (Tr8) measured in live embryos at late stage 17 using phase-contrast microscopy. Diameter values represent averages of at least 3 independent experiments.
For details about the transgenic strains or the origin of the mutant alleles, see methods.
n = number of embryos examined per genotype.
[b] ***, P < 0.001 in comparison to wt embryos.
[c] ***, P < 0.001 in comparison to *Ptp4E10D* embryos, unpaired two-tailed Student's *t*-test.
ns, P > 0.05 in comparison to *Ptp4E10D* embryos, unpaired two-tailed Student's *t*-test

Supplementary Table S2. The sequences of primers used for cloning.

Primers used for:	Primer name	Primer sequences (5'-3')
generating K554M mutation in Btk29A	Mutbtk p1	CTATCGACACAGCGGTCATGATGATGAAGGAAGGAACC
	Mutbtk p2	GGTTCCTTCCTTCATCATCATGACCGCTGTGTCGATAG
generating Y273D mutation in Wash	washY273D p1	GCATAGCGAATGATCTGCAAGACGAGCCAGTGGAGGAGCAAAC
	washY273D p2	GTTTGCTCCTCCACTGGCTCGTCTTGCAGATCATTCGCTATG
generating Y273F mutation in Wash	washY273F p1	CATAGCGAATGATCTGCAATTCGAGCCAGTGGAGGAGCAAAC
	washY273F p2	GTTTGCTCCTCCACTGGCTCGAATTGCAGATCATTCGCTATG
subcloning Btk29A into pJFRC28 vector	Btk primer1	GCTAATGCGGCCGCTTTAGTTTCGTATTAAATCGTTACTATTTAA
	Btk primer 2	GCTATTGGTACCCCTCCACCTCCGTCGGTTAGCGTTTGGGCAC
subcloning Ptp10D into pJFRC-MUH vector	Ptp primer1	GCTAATGCGGCCGCTGGGTGTGGTGAAGAGCGAA
	Ptp primer2	GCTATTGGTACCTATCCACCTCCTCTATCCGGTGCTGCATCCAC
subcloning Ptp10D into sJFRC28 vector	Ptpgfp primer1	GCTAATGCGGCCGCTGGGTGTGGTGAAGAGCG
	Ptpgfp primer2	GCTATTGGTACCGCTCCACCTCCTCTATCCGGTGCATCCAC
subcloning Wash to pJFRC-MUH	Not-wash-Kpn P1	ATTTTAGCGGCCGCATGGAGGAGTCACCTTACCTGCACAGC
	Not-wash-Kpn P2	ACTTTCGGTACCCTAGTTCCACCCATCCTCGTCGTCCTCGGAGTG
developing HA-Wash constructs	WashForwGATE	CACCATGGAGGAGTCACCTTACC
	WashRevGATEstop	CTAGTTCCACCCATCCTCGTCG
developing Btk29A-Flag constructs	BtkForwGATE	CACCATGATGGGCACTAAGCAT
	BtkRevGATEnon	GTCGGTTAGCGTTTGGGC
