# Supplementary material for: WASH phosphorylation balances endosomal versus cortical actin network integrities during epithelial morphogenesis
Source: Nat Commun. 2019 May 16;10:2193. doi: 10.1038/s41467-019-10229-6 (PMC6522504; doi:10.1038/s41467-019-10229-6)
Supplement: Supplementary file 3 — Reporting Summary [file 41467_2019_10229_MOESM3_ESM.pdf]

nature research

Corresponding author(s): Christos Samakovlis

Last updated by author(s): Apr 11, 2019

Reporting Summary

Nature Research wishes to improve the reproducibility of the work that we publish. This form provides structure for consistency and transparency in reporting. For further information on Nature Research policies, see Authors & Referees and the Editorial Policy Checklist.

Statistics

For all statistical analyses, confirm that the following items are present in the figure legend, table legend, main text, or Methods section.

n/a	Confirmed	
☐	☒	The exact sample size (n) for each experimental group/condition, given as a discrete number and unit of measurement
☐	☒	A statement on whether measurements were taken from distinct samples or whether the same sample was measured repeatedly
☐	☒	The statistical test(s) used AND whether they are one- or two-sided<br>*Only common tests should be described solely by name; describe more complex techniques in the Methods section.*
☒	☐	A description of all covariates tested
☒	☐	A description of any assumptions or corrections, such as tests of normality and adjustment for multiple comparisons
☐	☒	A full description of the statistical parameters including central tendency (e.g. means) or other basic estimates (e.g. regression coefficient) AND variation (e.g. standard deviation) or associated estimates of uncertainty (e.g. confidence intervals)
☐	☒	For null hypothesis testing, the test statistic (e.g. F, t, r) with confidence intervals, effect sizes, degrees of freedom and P value noted<br>*Give P values as exact values whenever suitable.*
☒	☐	For Bayesian analysis, information on the choice of priors and Markov chain Monte Carlo settings
☒	☐	For hierarchical and complex designs, identification of the appropriate level for tests and full reporting of outcomes
☒	☐	Estimates of effect sizes (e.g. Cohen's d, Pearson's r), indicating how they were calculated

Our web collection on statistics for biologists contains articles on many of the points above.

Software and code

Policy information about availability of computer code

Data collection	No Software was used
Data analysis	Graphpad Prism 6 was used to analyze and graph data for this work.

For manuscripts utilizing custom algorithms or software that are central to the research but not yet described in published literature, software must be made available to editors/reviewers. We strongly encourage code deposition in a community repository (e.g. GitHub). See the Nature Research guidelines for submitting code & software for further information.

Data

Policy information about availability of data

All manuscripts must include a data availability statement. This statement should provide the following information, where applicable:

- Accession codes, unique identifiers, or web links for publicly available datasets
- A list of figures that have associated raw data
- A description of any restrictions on data availability

NA

Field-specific reporting

Please select the one below that is the best fit for your research. If you are not sure, read the appropriate sections before making your selection.

☒ Life sciences ☐ Behavioural & social sciences ☐ Ecological, evolutionary & environmental sciences

For a reference copy of the document with all sections, see nature.com/documents/nr-reporting-summary-flat.pdf

October 2018

Life sciences study design

All studies must disclose on these points even when the disclosure is negative.

Sample size	No sample size calculation was performed in advance. The experimental unit of the study is considered the individual embryo. Empirically and based on our previous studies, sample sizes of 5-10 embryos are enough for revealing the type I error rate of rejecting the null hypothesis given that it is true.
Data exclusions	No data were excluded from the analysis.
Replication	All experiments presented were tested for reproducibility. We used 3-7 independent population of embryos or culture cells, collected on different days. Details are provided in figure legends or material and method section. We did not notice any limitations in reproducibility.
Randomization	Flies were grown in large populations, therefore embryos derive from thousands of mothers. When we picked up embryos to analyze, only the following criteria were used: the correct genotype and developmental stage. Otherwise, all samples were randomly allocated.
Blinding	The blinding was not possible when picking up the correct genotypes and developmental stages of embryos.

Reporting for specific materials, systems and methods

We require information from authors about some types of materials, experimental systems and methods used in many studies. Here, indicate whether each material, system or method listed is relevant to your study. If you are not sure if a list item applies to your research, read the appropriate section before selecting a response.

Materials & experimental systems

n/a	Involved in the study	
☐	☒	Antibodies
☐	☒	Eukaryotic cell lines
☒	☐	Palaeontology
☒	☐	Animals and other organisms
☒	☐	Human research participants
☒	☐	Clinical data

Methods

n/a	Involved in the study	
☒	☐	ChIP-seq
☒	☐	Flow cytometry
☒	☐	MRI-based neuroimaging

Antibodies

Antibodies used

For immunostaining, the following antibodies at the indicated dilutions were used:
mouse anti-Ptp10D (1:10, 8B22F5, Developmental Studies Hybridoma Bank, DSHB), rat anti-DE-Cad (1:50, DCAD2, DSHB),
mouse anti-Rab7 (1:100, DSHB),
mouse anti-Crb (1:10, Cq4, DSHB),
mouse anti-Coracle (1:100, C615.16, DSHB),
rabbit anti-GFP (1:400, A11122, Thermo Scientific),
chicken anti-GFP (1:400, abl3970, Abcam),
gp anti-Verm (Wang, Jayaram et al. 2006),
gp anti-Gasp (Tiklova, Tsarouhas et al. 2013),
rabbit anti-Uif (Chandrasekaran and Beckendorf 2005),
mouse anti-WASH (1:5, P3H3, DSHB),
mouse anti-FasIII (1:10, 7G10, DSHB),
rat anti-DE-Cad (1:50, DSHB).

For western blot analysis, the following primary antibodies were used at the indicated dilutions: rabbit anti-HA (1:2000, Abcam, ab9110), rabbit anti-FLAG (1:2000, Abcam, ab1162), mouse anti-FLAG M2 (1:3000, Sigma, F3165), rat anti-Btk29A (1:1000, (Tsikala et al., 2014)), mouse anti-Ptp10D (mixture of 8B22F5 and 45E10 antibodies used in 1:50 each, DSHB), mouse anti-WASH (1:10, P3H3, DSHB), rabbit anti-α-tubulin (1:2000, Cell signalling, 11H10), rabbit anti-PhosphoTyrosine (P-Tyr-1000, 1:2000, Cell Signaling Techonology, 8954), mouse polyclonal anti-Strumpellin (1:1000), mouse polyclonal anti-CCDC53 (1:1000) and mouse polyclonal anti-FAM21 (1:1000).

Validation

All the antibodies have been described, used and validated in previous studies.
However, when applicable, antibodies were verified for our system with antibody staining in mutant fly strains for the corresponding antigen protein.

Eukaryotic cell lines

Policy information about cell lines

Cell line source(s)	Drosophila Schneider's line 2 (S2) cells and Mouse Embryonic Fibroblasts (MEFs).
Authentication	The S2 cell line has been authenticated by the distributer (Drosophila Genomics Resource Center, DGRC) https://dgrc.bio.indiana.edu/cells/Catalog. MEFs line is described earlier (Gomez et al., MBofCell 2012).
Mycoplasma contamination	Cells were not tested for contamination of mycoplasma. However, viral or mycoplasma infections are rare phenomena for S2 cells derived from the DGRC.<br><br>(Cherbasa and Gonga, Methods, 2014)
Commonly misidentified lines<br>(See ICLAC register)	NA
