# Supplementary material for: WASH phosphorylation balances endosomal versus cortical actin network integrities during epithelial morphogenesis
Source: Nat Commun. 2019 May 16;10:2193. doi: 10.1038/s41467-019-10229-6 (PMC6522504; doi:10.1038/s41467-019-10229-6)
Supplement: Supplementary file 2 — Peer Review [file 41467_2019_10229_MOESM2_ESM.pdf]

Editorial Note: This manuscript has been previously reviewed at another journal that is not operating a transparent peer review scheme. This document only contains reviewer comments and rebuttal letters for versions considered at Nature Communications.

Reviewers' comments:

Reviewer #1 (Remarks to the Author):

In this paper, the authors present phenotypic data showing that actin regulation mediated by Wash and tyrosine kinases is required for normal trachea development. They also present biochemical data showing that Wash is phosphorylated by Btk29A. While the phenotypic and biochemical data have been improved in response to previous comments, the link between these remains weak.

The authors argue that Arp2/3 function regulated by WASH is important for the endocytic pathway. While phenotypic data by overexpressing Y261 point mutations or a VCA-truncated form of WASH is consistent with this idea, is the same phenotype obtained when Arp2/3 itself is knocked down? In addition, WASH works in concert with the WASH Regulatory Complex. Drosophila and mammalian studies have shown that the expression of these other complex members is lost in a WASH mutant background (Jia 2010 PNAS; Derivery 2009 Dev Cell; Verboon 2018 J Cell Sci). These point mutations may be disrupting the WASH's interaction with its regulatory complex. Can Arp2/3 and members of the WASH Regulatory Complex be immunoprecipitated from WashY261 lysates?

It was recently shown that the WASH related actin nucleation promoting factor SCAR can be upregulated in Drosophila WASH mutants to rescue its oogenesis defects (Verboon 2018 J Cell Sci). WASP is known to be activated by Src kinase. Is WASP expression altered in their mutant phenotypes? The authors show that re-expression of btl>WASH in WASH mutants restored the luminal clearance phenotype of WASH mutants. Can similar re-expression of WASP rescue this phenotype as well?

Minor points
1) Figure 4g-h. If WASH act as downstream of PTPs and Btk29A, the 4E10D double mutant and the 4ED10 wash185 triple mutant would be expected to exhibit similar phenotypes since WASH function is already disrupted in the PTPs double mutant background. To strengthen the possibility of parallel functions, the authors should show the phenotype of wash185 mutant alone in this assay.

2) Table 1. The statistical significance between the wild type (w1118) and 4E10D embryos should not be "ns". Similarly, since 4E10D is used as a reference, the second line with 4E10D should not be "ns".

Reviewer #2 (Remarks to the Author):

This manuscript by Tsarouhas et al. reports a novel regulatory mechanism of endocytic pathway involving endosomal actin polymerization factor WASH activated by tyrosine kinase Btk29A. In the previous version, the authors showed in the Drosophila trachea this pathway is negatively regulated by receptor tyrosine phosphatases and activation of this pathway leads to rapid disassembly of cortical F-actin cables and massive wave of endocytosis that is crucial for maturation of the tracheal tubule.

The revised manuscript includes new data on the association of Ptp10D and Btk29A, association of WASH with Btk29A and its tyrosine phosphorylation. Using a mouse cell model, they showed WASH activity is required for its association with F-actin. The new data strongly supports the molecular basis of this regulatory pathway where association of Btk29A with WASH and its phosphorylation at the key tyrosine residue of WASH. Overall, this work reports an interesting case of F-actin regulation coupled with endocytic pathway activation in the important model system of tube morphogenesis. Although precise molecular mechanism of what triggers this series of events and how cortical F-actin cables interfere endocytosis remains to be addressed, this work in its present form provides significant advance that would be of interest to the wide range of scientists in the field of cell and developmental biology.

Minor comments:
Figure 8 b,c, Fig. S8e: The quantification methods for those data were not found in the "Material and Methods" section.

Figure S6: The overexpression of DAAM[C], which is thought to have a dominant negative effect, potentially has an effect on the secretion of luminal ANF-GFP before the clearance. Please confirm if this is the case or not.

P12 line 372. "This genetic analysis indicates that PTPs control the activity of Arp2/3 through Wash." The genetic interaction data are not strong enough to support this statement since there remains a possibility that wash-Arp2/3 and PTPs-Arp2/3 work in parallel. Further experiments such as genetic interaction of 3 components (PTPs, wash, Arp2/3) is needed to make this statement. Otherwise, the statement should be modified.

Reviewer #3 (Remarks to the Author):

This is a significantly revised version of a previously reviewed paper that investigates the mechanisms by which mutations in the receptor tyrosine phosphatases Ptp10D and Ptp4E cause premature clearing of luminal proteins and disassembly apical actin bundles. Normally, luminal clearing results from activation of a massive wave of endocytosis, and the authors present convincing evidence that the absence of Ptp10D and Ptp4E activity results in the premature onset of the bulk endocytic that can be suppressed by mutations in genes controlling endocytosis, the non-receptor tyrosine kinase btk and the actin nucleation regulator, WASH. Further key observations include that Btk29A can be co-immunoprecipitated with Ptp10D and WASH and that Btk29A phosphorylates WASH on a tyrosine conserved to humans. This phosphorylation is critical for Drosophila WASH function and for association of WASH with endosomes in mammalian cells. Moreover, a phospho-mimetic WASH induces premature endosome actin accumulation, luminal endocytosis and cortical F-actin disassembly. The authors then present a model in which WASH activity decides the balance between structural actin assembly and actin being used in endocytosis.

As WASH is an important regulator of actin nucleation and endocytosis, the results will be of interest to many researchers. The paper advances the field in several important ways. First, the authors identify WASH as playing a central role in the switch between stable actin bundles that buttress cell shape and actin being used for endocytosis. Second, The authors identify btk as the kinase regulating WASH in this role. Third, the authors identify a conserved tyrosine in WASH that they show is in require for regulation of endocytosis in Drosophila and for endocytic association in mammals. Previously, WASH has not been identified as a Btk substrate, and has barely been reported as a Src-family kinase substrate. Fourth, this role of PTPs, Btk and WASH in switching actin from being in a supracellular cytoskeletal support system to underpinning a massive wave of endocytosis, occurs in the context of a developing tissue. These phenomena would unlikely to be discovered in tissue culture cells since such a complicated organ-level process is unlikely to be recapitulated in vitro. Moreover, the critical analyses are performed in vivo, which would be difficult, to say the least, in a mammalian system. Thus, this work is notable for revealing in vivo roles of PTPs, Btk and WASH that would be unlikely to be identified any other way.

The work is generally well done, and the writing generally clear (both subject to a few points below). Importantly, the authors have resolved almost all of the extensive comments of the previous reviews. Thus, subject to the comments below, I feel this article is appropriate for Nature Communications.

Major comment:

I am loath to ask for further work after a paper has been reviewed before, but this is an important issue and was only partially resolved in respond to a previous reviewer's comments. Reviewer 1 commented that the colocalization of WASH with Rab7-GFP was not 100% or even close to that. The authors responded by qualifying their language, but the more fundamental issue is that it is hard to assess the overlap WASH or Ptps and any of the Rabs.
In fig. S2a, the authors say that:
"we detected cytoplasmic Ptp10D-positive puncta co-stained with YRab5, YRab7 and weaker with YRab11 (Supplementary Fig. 2a)"

However, there is so little co-localization of Ptp10D and any of these Rabs that is unclear if the co-localization with YRab-11 is in fact weakest or pretty much the same. For the data in Fig. S2a to be discussed the authors need to 1) quantify the amount of overlap with any given Rab and 2) show and quantify images of a Rab (or other random punctate cytosolic cell structure) that doesn't overlap with WASH so that there is a basis to compare overlapping and non-overlapping.

Along those lines, the authors say that:

Staining with an anti-WASH antibody showed a punctate distribution in the airways and co-localization with the endosomal marker Rab7 during the period of luminal
clearance (Fig. 4f)

For this statement to be believable I would like to see controls for this experiment, including quantification of the co-localization, and how that changes over time.

Minor comments:

- Reviewer 1's first minor comment was that they found in the paper that the switching between embryonic stages and time to be confusing. The reviewer requested a timeline be put in Figure 1. The authors added the timeline to fig. S1. Minimally, I agree with Reviewer 1 and think the conversion timeline should be in the main Fig 1, not the supplementary. Better would be to at least label Fig 1a with developmental time. E.g. "st. 16 (16h)" instead of just "st. 16". And best would be to label more panels and figure with both .

- Lines 177-179: It would be very helpful for the authors to briefly describe how this reporter works. For most readers, they will have to look up the reference before being able to understand what the reporter is showing, or how it might be fooled.

- Lines 219-220: "Re-expression of either Btk29A isoforms specifically in the mutant airways led to a significant restoration of ANF-GFP protein clearance but did not rescue the gas-filling defect (Fig. 3h and Supplementary Fig. 3g). These observations indicate that Btk29A kinase activity is required in the airways for initiation and completion of luminal material clearance." They follow this with "Btk29A has an additional non-tracheal function required for airway gas filling 30.", implying that that incomplete rescue is due to the non-tracheal function of btk. However, an equally plausible explanation is that the two isoforms of btk exist for a reason and do not have identical functions such that complete rescue would require simultaneous rescue with both isoforms. This possibility is consistent with the partial rescue observed in figure 3 h. The authors' do not need to do the simultaneous expression experiment, but they are encourage to comment on this in the text or discussion.

- Rebuttal to point 1 of the first reviewer comments: The authors state "According to reviewer, "WASH is a well-known target of Btk29A" and "Src kinase is also known to act on WASH" (comment 2). We

have looked extensively in the literature and we haven’t been able to find any such data. WASH is not known to be a target of Btk and thus we strongly believe that also this result presents a novel finding.

While I also cannot find evidence in the literature that WASH is a well-known target of Btk, there is one recent (2016) paper showing the WASH is a substrate of the Lck Src-family kinase (Huang L, et al. Cell Death Dis. 2016) Nonetheless, I agree with the authors that Btk phosphorylating WASH is a novel point about their paper.

Reviewers' comments:

Reviewer #1 (Remarks to the Author):

In this paper, the authors present phenotypic data showing that actin regulation mediated by Wash and tyrosine kinases is required for normal trachea development. They also present biochemical data showing that Wash is phosphorylated by Btk29A. While the phenotypic and biochemical data have been improved in response to previous comments, the link between these remains weak.

The authors argue that Arp2/3 function regulated by WASH is important for the endocytic pathway. While phenotypic data by overexpressing Y261 point mutations or a VCA-truncated form of WASH is consistent with this idea, is the same phenotype obtained when Arp2/3 itself is knocked down?
In addition, WASH works in concert with the WASH Regulatory Complex. Drosophila and mammalian studies have shown that the expression of these other complex members is lost in a WASH mutant background (Jia 2010 PNAS; Derivery 2009 Dev Cell; Verboon 2018 J Cell Sci). These point mutations may be disrupting the WASH's interaction with its regulatory complex. Can Arp2/3 and members of the WASH Regulatory Complex be immunoprecipitated from WashY261 lysates?

We followed this suggestion and generated *Arpc1*^{Q25sd} mutant flies carrying *btl*>ANF-GFP and we examined the luminal protein clearance in embryos. Similar to *wash*^{185}, *Arpc1*^{Q25sd} mutant embryos failed to clear luminal ANF-GFP. This is consistent with the proposed model that Arp2/3 function is regulated by WASH during endocytotic clearance. The new data are included in Supplementary Fig. 8j.

We also compared the interactions of SHRC members with WASH and the WASH^{Y273D} version, which disrupts the cortical network and tips the balance towards endosomal actin assembly. We understand that the reviewer is worried that the dominant effects caused by the WASH^{Y273D} may be due to some neomorphic effect not related to the normal functions of WASH on endosomal actin. We therefore, used immunoprecipitations in *Drosophila* S2 cells to examine the interactions of these constructs with members of the WASH Regulatory Complex. Both HA-tagged WASH and HA-WASH ^{Y261D} can immunoprecipitate the members of the WASH regulatory complex, strumpellin and CCDC53. We also showed in complementary experiments that immunoprecipitation with an anti- CCDC53 antibody (Verboon, 2015 MBoC and Verboon 2018 J Cell Sci) can bring down similar amounts of HA-WASH and WASHY^{273D}. These data are included in Supplementary Figure S8 (k, i) and indicate that the WASHY^{273D} mutation does not disrupt the interactions of WASH with its regulatory complex (Page 11, line 27). Accordingly, we suggest that the dominant phenotypes caused by WASH^{Y273D} are not due to aberrant binding to the WASH regulatory complex.

It was recently shown that the WASH related actin nucleation promoting factor SCAR can be upregulated in *Drosophila* WASH mutants to rescue its oogenesis defects (Verboon 2018 J Cell Sci). WASP is known to be activated by Src kinase. Is WASP expression altered in their mutant phenotypes? The authors show that re-expression of btl>WASH in WASH mutants restored the luminal clearance phenotype of WASH mutants. Can similar re-expression of

WASP rescue this phenotype as well?

We had shown that *Src42A* and *Src64B* mutations do not influence the tube shape and luminal clearance phenotypes of PTP mutants, whereas *Btk29A* mutations do. Additionally, *wash* and *Btk29A* mutants show similar luminal clearance defects regardless of a potential redundant function of WASP in the absence of Wash. We believe that the potential compensatory mechanism suggested by the reviewer, where WASP maybe upregulated in the absence of WASH is interesting but beyond the scope of this manuscript, which is focused on the regulation of WASH.

Minor points
1) Figure 4g-h. If WASH act as downstream of PTPs and Btk29A, the 4E10D double mutant and the 4ED10 wash185 triple mutant would be expected to exhibit similar phenotypes since WASH function is already disrupted in the PTPs double mutant background. To strengthen the possibility of parallel functions, the authors should show the phenotype of wash185 mutant alone in this assay.

According to the reviewer´s suggestion, we added the analysis of tube shape phenotypes of *wash* mutants and compared to the *Ptp4E10D* phenotypes and *Ptp4E10D;wash* mutants (new figure 4g, g' and h). We did not detect a discernible phenotype in tube diameter in the airways of *wash* mutant. Because the phenotypes of this mutant (*wash*^{185}) in the airways (our results on lumen clearance, Fig 4a-c) and other organs are variable (Nagel et al., 2017 Cell Sci 130, 344-359, Verboon 2018 J Cell Sci) it is hard to draw any strong conclusions on the epistatic or parallel action of PTPs and Wash. Nevertheless, the results strengthen the notion that Ptps and wash may act in parallel as was suggested in the text (page 9 line 5).

2) Table 1. The statistical significance between the wild type (w1118) and 4E10D embryos should not be “ns”. Similarly, since 4E10D is used as a reference, the second line with 4E10D should not be “ns”.

We agree with the reviewer. We corrected the mistake in the current version.

Reviewer #2 (Remarks to the Author):

This manuscript by Tsarouhas et al. reports a novel regulatory mechanism of endocytic pathway involving endosomal actin polymerization factor WASH activated by tyrosine kinase Btk29A. In the previous version, the authors showed in the Drosophila trachea this pathway is negatively regulated by receptor tyrosine phosphatases and activation of this pathway leads to rapid disassembly of cortical F-actin cables and massive wave of endocytosis that is crucial for maturation of the tracheal tubule.

The revised manuscript includes new data on the association of Ptp10D and Btk29A, association of WASH with Btk29A and its tyrosine phosphorylation. Using a mouse cell model, they showed WASH activity is required for its association with F-actin. The new data strongly supports the molecular basis of this regulatory pathway where association of Btk29A with WASH and its phosphorylation at the key tyrosine residue of WASH. Overall, this work reports an interesting case of F-actin regulation coupled with endocytic pathway activation in the important model system of tube morphogenesis. Although precise molecular mechanism of what triggers this series of events and how cortical F-actin cables interfere endocytosis

remains to be addressed, this work in its present form provides significant advance that would be of interest to the wide range of scientists in the field of cell and developmental biology.

Minor comments:
Figure 8 b,c, Fig. S8e: The quantification methods for those data were not found in the "Material and Methods" section.

We have added the information on quantification in the Material and Methods section.

Figure S6: The overexpression of DAAM[C], which is thought to have a dominant negative effect, potentially has an effect on the secretion of luminal ANF-GFP before the clearance. Please confirm if this is the case or not.

We have analyzed the secretion of luminal ANF-mCherry in the tracheal overexpression of DAAM^{C}. Secretion of luminal ANF-mCherry is not affected in *btl*>DAAM^{C} embryos. This is added in the text page 10, line 1.

P12 line 372. "This genetic analysis indicates that PTPs control the activity of Arp2/3 through Wash." The genetic interaction data are not strong enough to support this statement since there remains a possibility that wash-Arp2/3 and PTPs-Arp2/3 work in parallel. Further experiments such as genetic interaction of 3 components (PTPs, wash, Arp2/3) is needed to make this statement. Otherwise, the statement should be modified.

We fully agree with the reviewer that this sentence is misleading. We had written in page 8 line 30 (Page 9, 5-6 in current version) "that PTP and WASH may act in parallel" and then contradicted ourselves in page 12. We clarified accordingly in page 12 line 372 (page 13, line 1-2, current version) to indicate that PTP and WASH may act in parallel.

Reviewer #3 (Remarks to the Author):

This is a significantly revised version of a previously reviewed paper that investigates the mechanisms by which mutations in the receptor tyrosine phosphatases Ptp10D and Ptp4E cause premature clearing of luminal proteins and disassembly apical actin bundles. Normally, luminal clearing results from activation of a massive wave of endocytosis, and the authors present convincing evidence that the absence of Ptp10D and Ptp4E activity results in the premature onset of the bulk endocytic that can be suppressed by mutations in genes controlling endocytosis, the non-receptor tyrosine kinase btk and the actin nucleation regulator, WASH. Further key observations include that Btk29A can be co-immunoprecipitated with Ptp10D and WASH and that Btk29A phosphorylates WASH on a tyrosine conserved to humans. This phosphorylation is critical for Drosophila WASH function and for association of WASH with endosomes in mammalian cells. Moreover, a phospho-mimetic WASH induces premature endosome
actin accumulation, luminal endocytosis and cortical F-actin disassembly. The authors then present a model in which WASH activity decides the balance between structural actin assembly and actin being used in endocytosis.
As WASH is an important regulator of actin nucleation and endocytosis, the results will be of interest to many researchers. The paper advances the field in several important ways. First, the authors identify WASH as playing a central role in the switch between stable actin

bundles that buttress cell shape and actin being used for endocytosis. Second, The authors identify btk as the kinase regulating WASH in this role. Third, the authors identify a conserved tyrosine in WASH that they show is in require for regulation of endocytosis in Drosophila and for endocytic association in mammals. Previously, WASH has not been identified as a Btk substrate, and has barely been reported as a Src-family kinase substrate. Fourth, this role of PTPs, Btk and WASH in switching actin from being in a supracellular cytoskeletal support system to underpinning a massive wave of endocytosis, occurs in the context of a developing tissue. These phenomena would unlikely to be discovered in tissue culture cells since such a complicated organ-level process is unlikely to be recapitulated in vitro. Moreover, the critical analyses are performed in vivo, which would be difficult, to say the least, in a mammalian system. Thus, this work is notable for revealing in vivo roles of PTPs, Btk and WASH that would be unlikely to be identified any other way.
The work is generally well done, and the writing generally clear (both subject to a few points below). Importantly, the authors have resolved almost all of the extensive comments of the previous reviews. Thus, subject to the comments below, I feel this article is appropriate for Nature Communications.

Major comment:

I am loath to ask for further work after a paper has been reviewed before, but this is an important issue and was only partially resolved in respond to a previous reviewer’s comments. Reviewer 1 commented that the colocalization of WASH with Rab7-GFP was not 100% or even close to that. The authors responded by qualifying their language, but the more fundamental issue is that it is hard to assess the overlap WASH or Ptps and any of the Rabs.

In fig. S2a, the authors say that:
“we detected cytoplasmic Ptp10D-positive puncta co-stained with YRab5, YRab7 and weaker with YRab11 (Supplementary Fig. 2a)”

However, there is so little co-localization of Ptp10D and any of these Rabs that is unclear if the co-localization with YRab-11 is in fact weakest or pretty much the same. For the data in Fig. S2a to be discussed the authors need to 1) quantify the amount of overlap with any given Rab and 2) show and quantify images of a Rab (or other random punctate cytosolic cell structure) that doesn’t overlap with WASH so that there is a basis to compare overlapping and non-overlapping.

We followed the reviewer´s suggestion. We quantified the colocalization of intracellular puncta of Ptp10D with YRab5, YRab7, YRab11 and YRab1 (an endocytotic unrelated marker) by calculating the Pearson's correlation coefficient *r*. In total, 1123 intracellular Ptp10D puncta in 36 embryos were analyzed. The degree of colocalization of Ptp10D puncta was significantly higher with YRab5 or YRab7, but not with Rab11, compared to control (YRab1). The quantification analysis supports our initial statement that Ptp10D intracellular puncta are preferentially co-stained with YRab5 or YRab7 and to a lesser extent with YRab11. We have added the data in Supplementary Figure 2b.

Along those lines, the authors say that:

Staining with an anti-WASH antibody showed a punctate distribution in the airways and co-localization with the endosomal marker Rab7 during the period of luminal clearance (Fig. 4f)

For this statement to be believable I would like to see controls for this experiment, including quantification of the co-localization, and how that changes over time.

We quantified the colocalization of intracellular puncta of WASH with YRab7 and YRab11 by calculating the Pearson's correlation coefficient *r*. In total, 680 puncta of WASH in 13 embryos were analyzed. The degree of colocalization of WASH puncta was significantly higher with YRab7, but not with Rab11, when compared to control (w1118 embryos). These data support the specificity of WASH punctate endosomal distribution and co-localization with Rab7 in the airways. Similar punctate distribution of WASH has been reported earlier in *Drosophila* airways (Dong et al., 2013, Nat. Commun.).

The colocalization of WASH puncta with YRab7 was significantly higher at later stages of tracheal development. These data included in the current version in Supplementary figure 4 (d & e).

Minor comments:

- Reviewer 1's first minor comment was that they found in the paper that the switching between embryonic stages and time to be confusing. The reviewer requested a timeline be put in Figure 1. The authors added the timeline to fig. S1. Minimally, I agree with Reviewer 1 and think the conversion timeline should be in the main Fig 1, not the supplementary. Better would be to at least label Fig 1a with developmental time. E.g. "st. 16 (16h)" instead of just "st. 16". And best would be to label more panels and figure with both.

We agree with the reviewer. We have added a graphical illustration of the timeline of *Drosophila* airway maturation in the Figure 1.

- Lines 177-179: It would be very helpful for the authors to briefly describe how this reporter works. For most readers, they will have to look up the reference before being able to understand what the reporter is showing, or how it might be fooled.

We agree with the reviewer. We have added more info in the text (Page 6, line 17-21).

We used a dual GFP-mCherry reporter of the *Drosophila* p62 orthologue Ref(2)P, to label ubiquitinated protein bodies destined for autophagic degradation (Nezis et al., 2008). Taking advantage of the differences in fluorescence stability between mCherry (pH>3.8) and GFP (pH>6.5) in low pH environments (ref), we could discriminate Ref(2)P localization in early endosomes, aggregates or inclusion bodies (green, green+magenta signals) with acidic compartments like autolysosomes and lysosomes (magenta signals). Expression of Ref(2)P-GFP-mCherry in the tracheal cells of *ptp4E10D* mutants showed a relative increase of the mCherry (magenta) signals accompanied by a simultaneous reduction of GFP intensity compared to wild type. These results indicate that acidic compartments (i.e lysosomes) are more frequent in *ptp4E10D* mutant tracheal cells than in their wild type counterparts (Supplementary figure 2c).

- Lines 219-220: “Re-expression of either Btk29A isoforms specifically in the mutant airways led to a significant restoration of ANF-GFP protein clearance but did not rescue the gas-filling defect (Fig. 3h and Supplementary Fig. 3g). These observations indicate that Btk29A kinase activity is required in the airways for initiation and completion of luminal material clearance.” They follow this with “Btk29A has an additional non-tracheal function required for airway gas filling 30.”, implying that that incomplete rescue is due to the non-tracheal function of btk. However, an equally plausible explanation is that the two isoforms of btk exist for a reason and do not have identical functions such that complete rescue would require simultaneous rescue with both isoforms. This possibility is consistent with the partial rescue observed in figure 3 h. The authors’ do not need to do the simultaneous expression experiment, but they are encourage to comment on this in the text or discussion.

We re-phrased the text to avoid any miss-understanding. Re-expression of either Btk29A isoforms specifically in the mutant airways led to a significant restoration of ANF-GFP protein clearance but did not rescue the gas-filling defect (Fig. 3h and Supplementary Fig. 3g). These observations indicate that Btk29A kinase activity is required in the airways for initiation and completion of luminal material clearance. The failure to rescue the gas filling phenotype can be explained by the requirement of Btk29A in posterior spiracle morphogenesis. These structures are also required for gas filling but do not express *btl*Gal4 [34]. Alternatively, both isoforms may be required for gas filling in the airways after protein clearance. We have revised the text (Page 7, line 33, 34 & Page 8 line 1).

- Rebuttal to point 1 of the first reviewer comments: The authors state “According to reviewer, “WASH is a well-known target of Btk29A” and “Src kinase is also known to act on WASH” (comment 2). We have looked extensively in the literature and we haven’t been able to find any such data. WASH is not known to be a target of Btk and thus we strongly believe that also this result presents a novel finding.

While I also cannot find evidence in the literature that WASH is a well-known target of Btk, there is one recent (2016) paper showing the WASH is a substrate of the Lck Src-family kinase (Huang L, et al. Cell Death Dis. 2016) Nonetheless, I agree with the authors that Btk phosphorylating WASH is a novel point about their paper.

REVIEWERS' COMMENTS:

Reviewer #1 (Remarks to the Author):

This paper describes antagonistic regulatory mechanisms for F-actin in tracheal development. The authors have addressed all of the concerns raised in the initial view and the present version will be of interest to a wide readership.

Reviewer #3 (Remarks to the Author):

The paper is a revised version of a paper that had already been reviewed once. The authors' responses in terms of experiments and text additions satisfy the requests and comments of the reviewers. As this paper reports interesting new observations, it is now ready for publication.
